# Crucial Interactions between Altered Plasma Trace Elements and Fatty Acids Unbalance Ratio to Management of Systemic Arterial Hypertension in Diabetic Patients: Focus on Endothelial Dysfunction

**DOI:** 10.3390/ijms25179288

**Published:** 2024-08-27

**Authors:** Ines Gouaref, Amel Otmane, Mohamed Makrelouf, Samir Ait Abderrhmane, Ali El Mahdi Haddam, Elhadj-Ahmed Koceir

**Affiliations:** 1 Bioenergetics and Intermediary Metabolism Team, Laboratory of Biology and Organism Physiology, Biological Sciences Faculty, Nutrition and Pathologies Post Graduate School, Houari Boumediene University of Sciences and Technology (USTHB), Bab Ezzouar, Algiers 16123, Algeria; igouaref@gmail.com; 2 Tamayouz Laboratory, Centre de Recherche en Biotechnologie (CRBT), Ali Mendjli Nouvelle Ville UV 03 BP E73, Constantine 25000, Algeria; 3 Biochemistry and Genetics Laboratory, University Hospital Center, Mohamed Lamine Debaghine, Bab El Oued, Algiers 16000, Algeria; dr.otmane.am@gmail.com (A.O.); mmakrelouf@gmail.com (M.M.); 4 Diabetology Unit, University Hospital Center, Mohamed Seghir Nekkache (ex. HCA de Aïn Naâdja), Algiers 16208, Algeria; saitabderrahmane@yahoo.fr; 5 Diabetology Unit, University Hospital Center, Mohamed Lamine Debaghine, Algiers I-University, Bab El Oued, Algiers 16000, Algeria; haddam25@yahoo.fr

**Keywords:** systemic arterial hypertension (SAH), type 2 diabetes mellitus (T2DM), antioxidant trace elements, lipotoxicity, fatty acid ratio, endothelial dysfunction, oxidative stress, atherothrombogenic risk, insulin resistance, inflammation

## Abstract

The coexistence of SAH with T2DM is a common comorbidity. In this study, we investigated the link between altered plasma antioxidant trace elements (ATE: manganese, selenium, zinc, and copper) and fatty acids ratio (FAR: polyunsaturated/saturated) imbalance as transition biomarkers between vascular pathology (SAH) to metabolic pathology (T2DM). Our data revealed strong correlation between plasma ATE and FAR profile, which is modified during SAH-T2DM association compared to the healthy group. This relationship is mediated by lipotoxicity (simultaneously prominent visceral adipose tissue lipolysis, significant flow of non-esterified free fatty acids release, TG-Chol-dyslipidemia, high association of total SFA, palmitic acid, arachidonic acid, and PUFA ω6/PUFA ω3; drop in tandem of PUFA/SFA and EPA + DHA); oxidative stress (lipid peroxidation confirmed by TAS depletion and MDA rise, concurrent drop of Zn/Cu-SOD, GPx, GSH, Se, Zn, Se/Mn, Zn/Cu; concomitant enhancement of Cu, Mn, and Fe); endothelial dysfunction (endotheline−1 increase); athero-thrombogenesis risk (concomitant rise of ApoB_100_/ApoA_1_, Ox-LDL, tHcy, and Lp(a)), and inflammation (higher of Hs-CRP, fibrinogen and ferritin). Our study opens to new therapeutic targets and to better dietary management, such as to establishing dietary ATE and PUFA ω6/PUFA ω3 or PUFA/SFA reference values for atherosclerotic risk prevention in hypertensive/diabetic patients.

## 1. Introduction

In 2023, the World Health Organization established a report that highlights that the incidence of systemic arterial hypertension (SAH) would be responsible for 17 million deaths per year [1]. SAH is often a comorbidity associated with type 2 diabetes mellitus (T2DM), having the cardiometabolic syndrome (CMS) in common. The increase in chronic SAH and T2DM related to cardiovascular mortality is attributable to aggravating of CMS risk factors, but with similarities and differences [2]. SAH is primarily metabolic and vascular disorders, recognized as ischemic heart failure and stroke, mainly increased triglycerides and cholesterol, visceral adiposity, fasting glucose intolerance, and elevated blood pressure (BP). Insulin resistance remains the major pivotal factor of CMS in both SAH and T2DM [3].

Between 2000 and 2019, epidemiological statistics from the GBD (global burden of metabolic disease) study show an increase in the mortality level for T2DM and SAH linked to dyslipidemia and hyperglycemia, which generate glucolipotoxicity and a vascular hemodynamic disorder in diabetic β-cell damage [4] and hypertension [5], intimately interfering with endothelial function [6]. The interaction between SAH and T2DM is multifactorial complexity related to endothelial dysfunction and the athero-thromboembolic process, which represents the major contributor to ischemic heart disease and myocardial infarction [7]. Several studies have shown that interactions between dyslipidemia and lipotoxicity are closely influenced by fatty acid (FA) ratio, mainly polyunsaturated/saturated FA (PUFA/SFA) unbalanced [8,9,10]. Furthermore, lipotoxicity-mediated endothelial dysfunction is caused by abnormally high levels of saturated FA, such as palmitic acid and cholesterol [11].

Recently, the American Health and Nutrition study was based on an examination survey that highlighted a strong correlation between dietary intake of SFA, and it has been shown that dietary ω-3 fatty acid deficiency contributes to the development of hypertension [12]. Randle demonstrated that plasma FA levels increase under fasting or T2DM and lead to insulin resistance by inhibition of carbohydrate oxidation [13]. Numerous studies have shown that in T2DM, the increase SFA to PUFA ratio was positively correlated with low-grade inflammation, insulinipenia, and atherosclerosis [14]. Some studies have revealed that alpha linolenic acid (C18: 3-n3) to linoleic acid (C18: 2-n6) ratio and docosahexaenoic (DHA) omega 3 (C22: 6-n3) to eicosapentaenoic (EPA) omega 3 (C20: 5-n3) ratio [15] have been extensively studied for their anti-atherosclerotic vascular benefits in T2DM [16].

In addition, PUFA−3 modulates blood pressure and regulates vascular hemodynamics by incorporating into the red blood cell membranes [17]. On the other hand, excess SFA in the bloodstream is recognized as predictive of coronary insufficiency [18] associated with venous thrombosis [19], mainly lauric (C12: 0), myristic (C14: 0), and palmitic (C16: 0). Moreover, several studies have shown that atherothrombotic events cause damage to endothelial function [20,21,22]. Furthermore, several studies have shown that atherothrombotic complications caused by lipids lead to endothelial dysfunction–oxidative stress interactions [23,24,25]. These events maintain other disorders related to plasma levels of oxidized low-density lipoprotein (Ox-LDL) and homocysteine. Indeed, hyperhomocysteinemia and Ox-LDL are associated with increased intima arteries damage to promote thrombosis by collagen activation pathway [26].

Moreover, Endothelin−1 (ET−1) has been associated with SAH, heart failure, and atherosclerosis. ET−1 is produced and released by the vascular endothelium. It is a potent endogenous vasoconstrictor peptide in SAH. [27]. The atherothrombogenic effect is also explained by the interaction between Ox-LDL and lipoprotein (a). It is interesting to note that omega 3 fatty acids correct this vascular deleterious effect [28]. Otherwise, atherothrombotic states are correlated with glucolipotoxicity–redox state interactions, which leads to oxidative stress (OS) and generates overproduction of reactive oxygen species (ROS) in SAH-T2DM comorbidity [29,30].

The involvement of sodium, potassium, and calcium in prevention and treatment of SAH-T2DM have been the major clinical studies investigations. The majority of studies involved dietary intervention by reducing sodium or potassium or calcium or supplementing them to reduce blood pressure. The studies were conducted in primary and secondary prevention compared to placebo or control in normotensive subjects or hypertensive patients.

Regarding sodium and potassium, dietary sodium and potassium intake are recognized to play a critical role in regulating blood pressure. High dietary salt intake may increase blood pressure, while a diet low in sodium and high in potassium is associated with lower blood pressure [31]. The effectiveness of sodium restriction or increased potassium intake in reducing the risk of hypertension has been described in clinical and observational research. The role of low sodium or high potassium intake modulates renin-angiotensin system activity, arterial stiffness, and endothelial dysfunction [32]. Some studies have shown that the sodium/potassium ratio is strongly correlated with blood pressure regulation, more so than sodium or potassium alone [33]. Some medical societies’ recommendations set a sodium intake limit of 2300 mg/day and 4680 mg/day for potassium intake [34]. The World Health Organization aims to reduce average sodium intake in the population by 30% by 2025 [35]. Moreover, the DASH diet (Dietary Approaches to Stop Hypertension) was designed to reduce sodium intake. This diet has proven effective in significantly lowering blood pressure in patients with stage 1 hypertension [36]. However, the correlation between low dietary sodium intake and low cardiovascular mortality has still not been found, making current knowledge on sodium very controversial [37].

Regarding calcium, the recent Cochrane database published three meta-analyses conducted on a cohort including a total of 20 clinical trials with 3512 participants to demonstrate that an increase in calcium intake slightly reduces systolic and diastolic blood pressure in normotensive people, suggesting a role in the prevention of hypertension. The effect on several predefined subgroups and a possible dose–response effect reinforces this conclusion. Even small reductions in blood pressure could have important health implications for reducing vascular disease. It is predicted that a 2 mm Hg lower systolic blood pressure would result in a reduction of about 10% in stroke mortality and about 7% in ischemic heart disease mortality. There is a great need for adequately powered clinical trials randomizing young people [38].

However, little attention has been given to the role of trace elements in SAH-T2DM etiology, mainly related to antioxidant trace elements (ATE) profile [39]. The metabolic balance between ATE is an important factor in homodynamic homeostasis by modulating the carbohydrates and lipid enzymes metabolism involved in the BP regulation [40,41] by influencing the cell membrane permeability via the vascular endothelial protection [42,43].

Plasma trace elements’ unsteadiness is strongly involved in SAH and T2DM, mainly selenium (Se), copper (Cu), zinc (Zn), and manganese (Mn). It is observed that the imbalance between ATE is associated with increased cardiovascular complications attributed to the therapeutic management difficulty [44]. On the other hand, fatty acid metabolism is regulated by ATE, particularly by zinc. Zn reduces the activity of Δ6 desaturases metabolizing linoleic acid to arachidonic acid [45]. Zn is involved as a regulator of the cardiovascular system and gastrointestinal lipid transport, prostaglandin metabolism, and cell membrane integrity [46]. Zn affects phosphodiesterase activity as an insulin-mimetic effect and regulates the lipolysis (fatty acids release) from adipose tissue [47]. Conversely, an increase in the plasma NEFA level disrupts the Zn binding to albumin since plasma albumin binds and transports both free fatty acids and Zn [48]. Also, Zn and Zn/Cu molar ratio are associated with the renin angiotensin–aldosterone system and lead to an elevation in systemic BP [49,50].

In regard to Cu, the deficiency of this trace element alters the saturated fatty acids to unsaturated fatty acids ratio. Indeed, this effect is exerted via the control of the expression of the genes involved in the synthesis of fatty acids and the metabolism of cholesterol, such as the SREBP−1 and SREBP−2 (sterol regulatory element binding proteins 1 and 2) genes, or the CYP7A1 gene encoding cholesterol 7-alpha hydroxylase in the liver [51,52]. SREBP−1 is specifically involved in regulating fatty acid synthesis, while SREBP−2 plays an important role in modulating cholesterol biosynthesis [53]. The SREBP−1c isoform is the main transcription factor used by insulin to activate the gene expression of lipogenic enzymes [54]. Studies involving rats have shown increased cholesterol levels in the body as a result of Cu deficiency [55]. Furthermore, Cu is correlated the activity of tyrosinase, a key enzyme in the norepinephrine synthesis (catecholamines), a major vasoconstriction neuromediator [56].

Manganese (Mn) is a trace element with an important role in endothelial function, lipid metabolism, and ROS destruction. Mn is involved in NO (nitric oxide) metabolism by its incorporation into the active site of arginine synthetase (urea cycle) [57,58]. Mn is implicated in the hepatic cholesterol and fatty acid regulation as a cofactor for mevalonate kinase and acetyl CoA carboxylase, respectively [59,60]. Mn is related to the AGEs (advanced glycation end products) [61]. Several studies have described that trace elements (Cu, Zn, and Mn) are strongly involved in the protection of hypertensive subjects via the active site of superoxide dismutase (SOD-Mn; SOD-Cu/Zn) [62,63].

Selenium (Se) is an essential trace element strongly involved in OS defense. Se has been integrated into the glutathione peroxidase active site in cellular antioxidant defense [64,65]. Se deficiency is described in Iran Se-endemic areas, known as cardiomyopathy Keshan disease. This pathology is characterized as necrosis and fibrosis of the myocardium and leads to shock and congestive heart failure. The main risk factors found were related to loss of GPx−1 activity [66]. The endothelium dysfunction linked to the ATE disorder and FA ratio imbalance is poorly described in the literature, and the mechanisms involved remain unsuccessfully elucidated in arterial hypertension with or without T2DM [67,68].

In this context, our investigation was conducted in four subject groups: healthy, diabetics without SAH, hypertensive without T2DM, and SAH–T2DM comorbidity. We sought to highlight interactions between CMS clusters, endothelium dysfunction, oxidative stress biomarkers related to the ATE profile, and fatty acid ratio imbalance, particularly PUFA/SFA-PUFA-n3/PUFA-n6 ratios in the management of essential hypertension in diabetic subjects.

In this study, we attempted to define the relationships between plasma trace elements with fatty acids profile and cardiovascular risk in human subjects with hypertension or T2DM or their combination using healthy subjects as controls. To our knowledge, very few previous studies have considered the relationship between fatty acid ratios and ATE profile including their ratio to prevent complications of hypertension in a diabetic subject.

## 2. Results

### 2.1. Clinical Characterization According Cardiometabolic Syndrome of Cohort Study

The sex/gender data are mentioned in Table 1. In this study, we noticed during our clinical investigation, the percentage of T2DM women participants is higher than men participants (53% versus 47%, respectively, *p* < 0.001). Contrarily, hypertension is more prevalent in men participants than in women participants (71% versus 29%, respectively, *p* < 0.001). Interestingly, we observed that group IV (hypertensive–diabetic participants) is represented predominantly by women participants compared to men participants (64.5% versus 35.5%, respectively, *p* < 0.001). The anthropometric data are summarized in Table 1. A relationship was observed between body mass index (BMI) and body fat percentage (BF), but not with body weight. The waist circumference (WC) traduced an abdominal adipose tissue depot, reflecting that visceral adiposity is significantly increased (*p* < 0.001) in participant groups II (Diabetics), III (Hypertensive), and IV (Hypertensive–diabetic) compared to group I (Healthy). A positive and significant correlation was observed between WC and BMI in groups II, III, and IV versus group I (r = +0.88, *p* < 0.001). The WC/Waist Hips ratio and the BF mass percentage highlight an adipose tissue accretion in abdominal-trunk topographic in male participants, which corroborate the android obesity, in contrast to a fat abdominal-ileal accumulation in female participants, indicating the gynoid obesity profile. This correlation was confirmed in all participants of groups II and IV versus group I. A strongly positive connection was established between BF and the WC/Waist Hips ratio in groups II and IV versus group I, but not with group III (r = +0.69).

An insulin resistance state (IR) is revealed in groups II and III and becomes more prominent in the IV group participants versus group I (Table 2). The Homa-IR index is increased by 192% and 374% in the hypertensive groups (II and IV) versus the control group, respectively (*p* < 0.0001). The correlation is positive between SBP and the Homa-IR index in the hypertensive groups (r = +0.58; r = +0.47, respectively). Concomitantly with IR state, hyperinsulinism is observed in all groups compared to the healthy group (Table 2). Fasting plasma insulin levels are increased by 88% in the III group and become bursting in the IV group (+159%) vs. the control group (*p* < 0.0001). The hyperinsulinism state is positively correlated with the Homa-IR index, WC, and % of BF (r = +0.63; r = +0.71 and r = +0.94, respectively).

**Figure 1:**
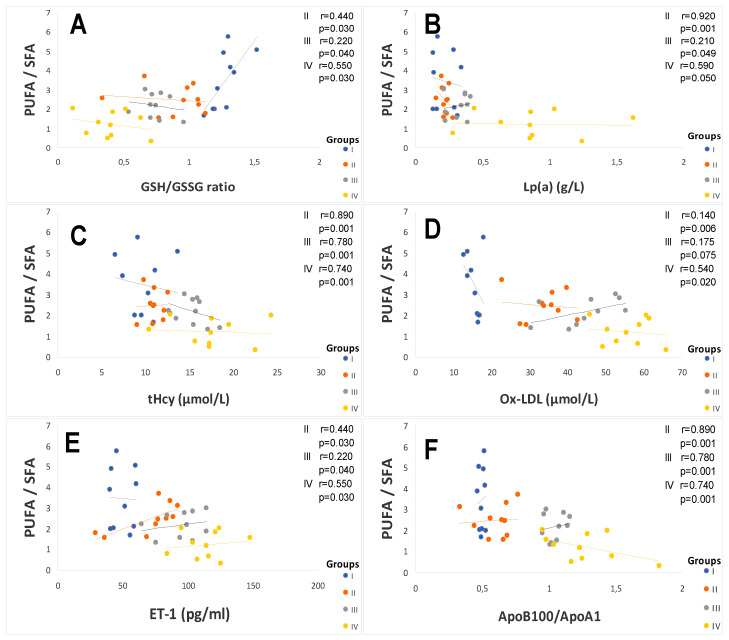
Pearson correlation between PUFA/SFA ratio and GSH/GSSG ratio (**A**), Lp (a) (**B**), tHcy (**C**), Ox-LDL (**D**), ET−1 (**E**), and ApoB100/ApoA1 (**F**) plasma levels in different trial groups. Group I: Healthy control participants; Group II: Diabetic participants; Group III: Hypertensive participants; Group IV: Hypertensive–diabetic participants. PUFA: polyunsaturated fatty acids; SFA: saturated fatty acids. GSH/GSSG: reduced glutathione/oxidised glutathione; Lp (a): Lipoprotein (a); tHcy: total homocysteine; Ox-LDL: Oxidized Low-Density Lipoprotein; ET−1: Endothelin 1; ApoB100/ApoA1: Apolipoprotein B100/Apolipoprotein A1.

The metabolic parameters data mentioned in Table 2 indicate that subjects in group III remained normoglycemic, on average 5.33 ± 0.81 mmol/L (0.96 ± 0.15 g/L) of fasting plasma glucose despite their abdominal adiposity. In contrast, in group IV, although they are treated with metformin (2 to 3 g of Glucophage/day), they persist as hyperglycemic, on average 9.63 ± 0.66 mmol/L (1.73 ± 0.12 g/L). This reveals that the association of hypertension and diabetes aggravates hyperglycemia. These glycemic variations have a negative impact on HbA1C levels, reflecting long-term glycemic balance (6–8 weeks). The values recorded in Table 2 show that the HbA1C of group IV subjects is >7% vs. control group (*p* < 0.001), a sign of poor metabolic balance. However, we did not find a correlation between SBP and HbA1C in group IV. Patients with higher levels of endothelin−1 (ET−1) had a significantly greater cardiovascular risk in group IV (Table 2). Pearson correlation showed a very strong positive association between elevated plasma ET−1 endothelin levels and plasma concentrations of lipoprotein a (Lpa), homocysteine (Hcy), the ApoB_100_/ApoA_1_ ratio, and oxidized low-density lipoprotein (Ox-LDL) in group IV versus the healthy group (Figure 1).

**Figure 2:**
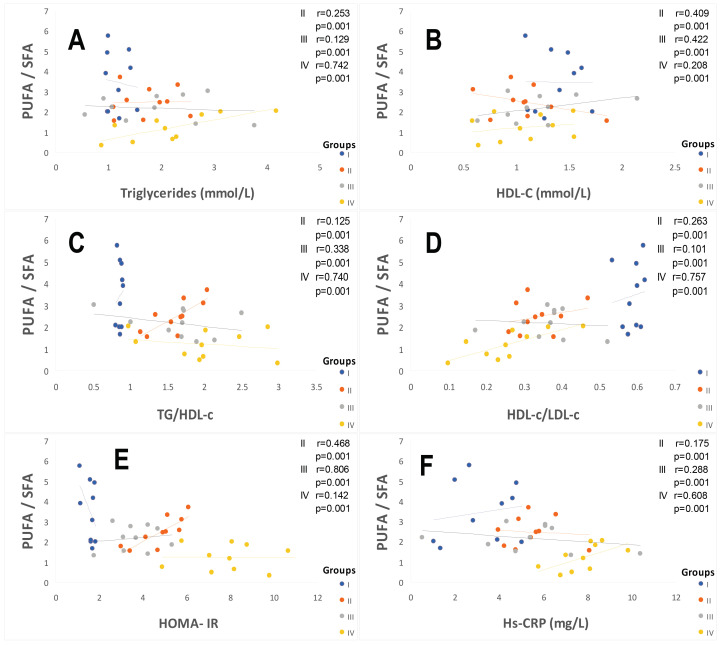
Pearson correlation between PUFA/SFA ratio and triglycerides (**A**), HDL cholesterol (**B**), triglycerides/HDL cholesterol ratio (**C**), HDL cholesterol/LDL cholesterol (**D**), HOMA-IR (**E**), and Hs-CRP (**F**) plasma levels in different trial groups. Group I: Healthy control participants; Group II: Diabetic participants; Group III: Hypertensive participants; Group IV: Hypertensive–diabetic participants. PUFA: polyunsaturated fatty acids; SFA: saturated fatty acids.

The lipid profile observed in group III and group IV patients is altered; it affects both triglyceride and cholesterol metabolism. If we examine the results recorded in Table 2, dyslipidemia is found both in triglyceride and cholesterol plasma levels in all patient groups compared to the control group. Hypercholesterolemia is marked by a significant HDL-c drop, whether in male or female subjects in group IV (Figure 2). The values of TG/HDL-C ratio increased to 50–56% in the hypertensive patients (group III and IV) vs. the control group (Table 3). TG/HDL-C concentration and visceral adiposity (WC/WH ratio) were highly correlated (*p* < 0.001; r = +0.99) in all groups II, III, and IV vs. the healthy group. Paradoxically, LDL-c concentrations remained normal (<4.14 mmol/L or <1.60 g/L) in all groups vs. the control group (Table 2). However, the HDL-c/LDL-c ratio drops drastically to 57% in hypertensive and diabetic patients’ groups versus healthy groups (*p* < 0.001, Table 3). The blood pressure values mentioned in Table 3 were increased on average by 24% in group IV compared to the control group. According to the World Health Organization criteria, groups IV and III are classified as grade I with moderate hypertension (140/90 to 159/99 mm Hg; systolic blood pressure/diastolic blood pressure, respectively). In contrast, group II remained in a normotensive state.

In this study, the atherothrombogenic risk was assessed by Lpa, Hcy, the ApoB_100_/ApoA_1_ ratio, and Ox-LDL. The data mentioned in Table 3 show that the Lp (a) levels are > 0.30g/L only in the hypertensive groups (III and IV) but not in the diabetic group compared to the healthy group. Furthermore, the plasma concentrations of Hcy are extremely high (>15 µmol/L) in the hypertensive–diabetic patients (*p* < 0.001), but moderate in the other groups. The ApoB/ApoAI ratio tended to be significantly higher in hypertensive patients (III and IV groups), but not in diabetic patients (II) vs. the control group (*p* < 0.001). The systemic lipid peroxidation was assessed by ox-LDL. The data mentioned in Table 3 show that ox-LDL levels are excessively higher in hypertensive patients and diabetic patients vs. healthy controls. The values are increased by 53%, 67%, and 72%, respectively, in groups II, III, and IV (*p* < 0.001). The Pearson correlation coefficient revealed a positive association between ox-LDL levels and saturated fatty acids (SFA), such as myristic and palmitic acid in group IV (Figure 1). In contrast, the correlation analysis showed a negative association between ox-LDL levels with omega−3 and omega−6 polyunsaturated fatty acids (PUFA), such as eicosapentaenoic acid and docosahexaenoic acid in group III (r = −0.694, r = −0.758; *p* < 0.001, respectively). It is interesting to note that higher tHcy and Lp (a) levels were positively correlated with significantly higher ox-LDL levels in group IV (r = +0.418, r = +0.509; *p* < 0.001). There was a significant positive correlation between hCys and selenium (r = +0.882, *p* < 0.0001).

Plasma levels of Hs-CRP and fibrinogen characterize the systemic inflammatory assessment and process in hypertensive and diabetic patients (Table 2). CRPus plasma levels were positively correlated with both plasma Lp(a) and Hcy concentrations in group IV (r = +0.971; r = +0.815; *p* < 0.0001). On the other hand, the correlation is positive between Hs-CRPus and % MGC (r = +0.617; *p* < 0.0001) and between Hs-CRPus and Homa-IR (r = +0.481; *p* < 0.001). There was no particular disorder in fibrinogen plasma levels in all groups.

### 2.2. Plasma Fatty Acids Profile

**Figure 3:**
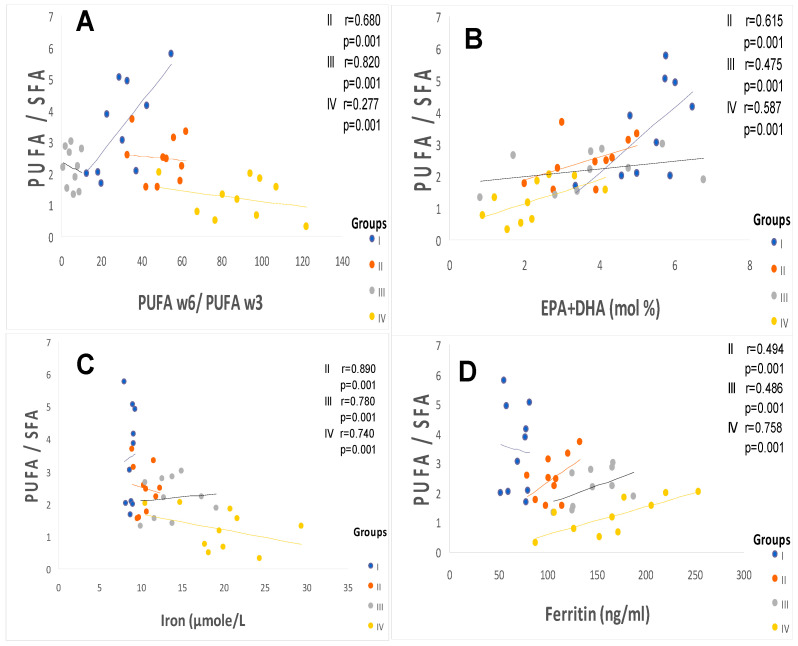
Pearson correlation between PUFA/SFA ratio and PUFAω3/PUFAω6 (**A**), EPA/DHA (**B**), iron (**C**), and ferritin (**D**) plasma levels in different trial groups. Group I: Healthy control participants; Group II: Diabetic participants; Group III: Hypertensive participants; Group IV: Hypertensive–diabetic participants. PUFA: polyunsaturated fatty acids; SFA: saturated fatty acids. EPA/DHA: fatty acids ω3 eicosapentaenoic acid (EPA) and fatty acids ω3 docosahexaenoic acid (DHA).

The saturated, monounsaturated, and polyunsaturated fatty acids data are summarized in Table 4. We noted that NEFAs (non-esterified free fatty acids) are moderately elevated in hypertensive patients and not altered in diabetic patients (+14%) vs. the control group. Indeed, NEFAs are extremely increased in group IV compared to group I (+40%; *p* < 0.001). Concurrently, total saturated fatty acids (SFA) are significantly higher in the group IV and III vs. the control group (+53%, +27%, respectively; <0.001) with a small change in group II (+16%). The plasma NEFAs profile disorder affects both saturated and unsaturated fatty acids. This concerns laurate (+65%), myristate (+145%), palmitate (+127%), and stearate (+71%). It should be noted that palmitate is the most dominant SFA in diabetic–hypertensive subjects (group IV). The fraction of monounsaturated fatty acids (MUFA), represented in our study by oleic acid (C18:1), is significantly reduced in group IV (−48% vs. control, *p* < 0.001), while it remains normal in the diabetic subjects (group II). Concerning polyunsaturated fatty acids (total PUFA), we did not observe any difference between group II and the hypertensive groups (III and IV) versus the control group. On the other hand, linoleic acid (PUFA-ω6) is moderately increased in group IV and group II vs. the control group (+29% and +24%, respectively); linolenic acid (3-PUFA) is half reduced in group IV (−48% vs. control group, *p* < 0.001) and moderately in group II (−17% vs. control). Likewise, arachidonic acid (PUFA-ω6) is depleted in all groups (−50% in group II vs. control group, *p* < 0.01), but more markedly in group IV (−62% vs. control group, *p* < 0.001). Regarding the ω3-PUFAs production resulting from the linolenic acid elongation, such as EPA (eicosapentaenoic acid) and DHA (docosahexaenoic acid), we recorded a drastic decrease in group IV vs. control group (−60%, *p* < 0.001) and moderate in group II and III vs. the control group (−30%). We also observed that group IV showed a significant increase in the linoleic acid (ω6 PUFA)/linolenic acid (ω3 PUFA) ratio associated with a marked decrease in the linolenic and arachidonic acids. Furthermore, we noticed that the PUFA/SFA ratio is significantly reduced in group IV (−48% vs. control group, *p* < 0.001) and no change in group II vs. the control group. At the same time, we noted an increase in the PUFA-ω6/PUFA-ω3 ratio and a reduction in the EPA (PUFA-ω3)/linolenic acid (PUFA-ω3) ratio in the two groups of patients (Figure 3). In group IV, we found a strong correlation between the fall in the PUFA/SFA ratio and the reduction in the HDL-c/LDL-c ratio (r = +0.660). This correlation was also associated with the increase in ApoB100/ApoA1, TG/HDL-c ratios, and Lp (a) concentrations in group IV (r = −0.550; *p* < 0.001). It is important to note the decrease in the PUFA/SFA ratio is negatively correlated with the increase in Ox-LDL levels in the hypertensive–diabetic group (r = −0.540; *p* < 0.020), but moderately in the hypertensive and diabetic group (Figure 1D).

### 2.3. Oxidative Stress Status

#### 2.3.1. Total Plasma Antioxidant Activity (TPAA) and Plasma Antioxidant Enzymatic Profile

**Figure 4:**
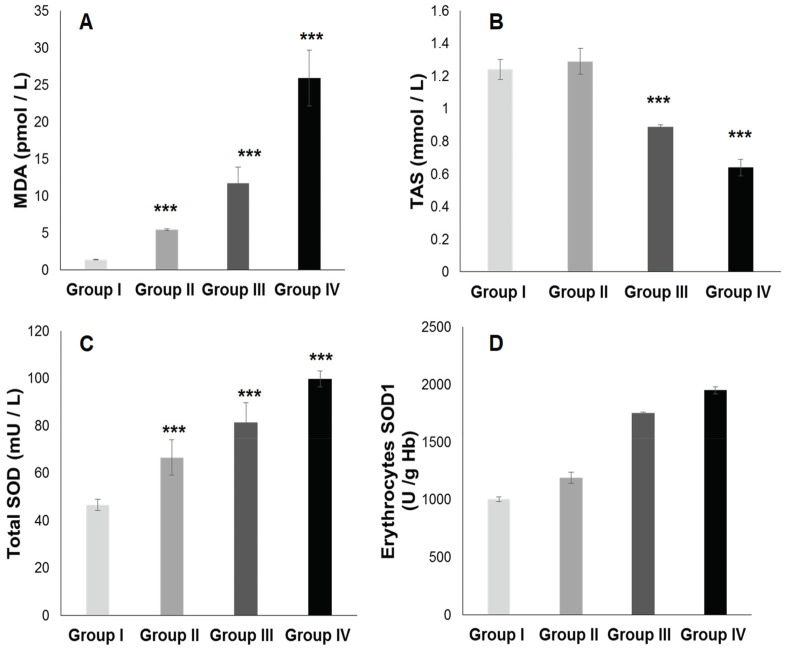
Total antioxidant status and SOD antioxidant activities in plasma of different trial groups. (**A**) MDA (malondialdehyde) levels; (**B**) TAS (total antioxidant status); (**C**) Total SOD (superoxide dismutase); (**D**) Erythrocytes SOD1. Group I: Healthy control participants; Group II: Diabetic participants; Group III: Hypertensive participants; Group IV: Hypertensive–diabetic participants. *** *p* < 0.001.

**Figure 5:**
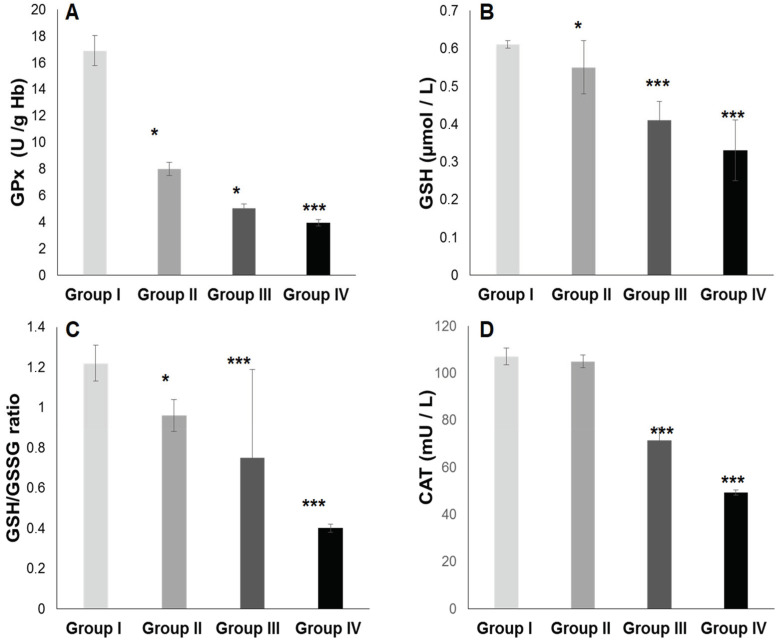
GPx (Glutathione peroxidase) and CAT (Catalase) antioxidant activities, and GSH (Glutathione) status in plasma of different trial groups. (**A**) GPx; (**B**) GSH; (**C**) GSH/GSSG (reduced glutathione/oxidized glutathione); (**D**) CAT. Group I: Healthy control participants; Group II: Diabetic participants; Group III: Hypertensive participants; Group IV: Hypertensive–diabetic participants. * *p* < 0.05; *** *p* < 0.001.

**Figure 6:**
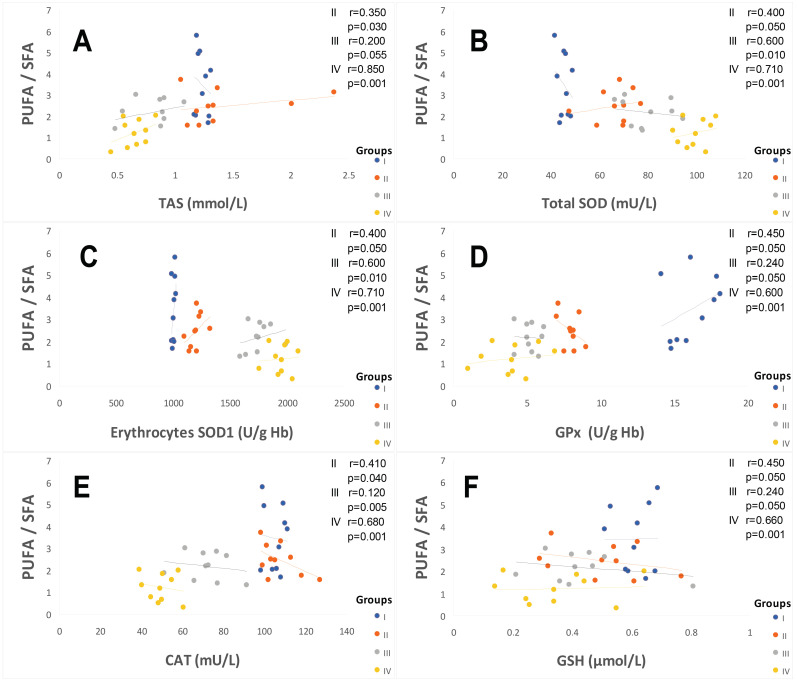
Pearson correlation between PUFA/SFA ratio and TAS (**A**), tSOD (**B**), eSOD1 (**C**), GPx (**D**), CAT (**E**), and GSH (**F**) plasma levels in different trial groups. Group I: Healthy control participants; Group II: Diabetic participants; Group III: Hypertensive participants; Group IV: Hypertensive–diabetic participants. PUFA: polyunsaturated fatty acids; SFA: saturated fatty acids. TAS: total antioxidant status; tSOD: total superoxide dismutase; eSOD1: erythrocyte superoxide dismutase−1; GPx: glutathione peroxidase; CAT: catalase; GSH: reduced glutathione.

The TPAA levels are significantly reduced only in hypertensive subject groups III and IV (Figure 4B), but moderately in diabetics group (II) compared to the control group (*p* < 0.001). The TPAA drop is inversely proportional to lipid peroxidation estimated by plasma TBARS levels and evaluated by MDA concentrations (Figure 4A), which are extremely high in hypertensive–diabetic patients (group IV). We also observed a significant increase in MDA levels in groups III and II compared to the control group (*p* < 0.0001). Paradoxically, total plasma SOD activity is strongly increased (Figure 4C) proportional to erythrocyte SOD activity (Figure 4D). As expected, plasma total GPx activity (Figure 5A) was significantly reduced in group IV, but moderately in groups III and II (*p* < 0.0001). Concomitantly, the total plasma catalase activity (Figure 5D) is significantly depleted in group III, but more marked in group IV (*p* < 0.0001). The hypertensive subjects (group IV and III) exhibit significant (*p* < 0.0001) depletion of plasma glutathione (GSH) levels compared to group II (Figure 5B). The plasma oxidized–reduced glutathione ratio (GSH/GSSG) is extremely reduced (Figure 5C) in hypertensive subjects (groups IV and III) compared to group II (*p* < 0.0001). The Pearson r coefficient summarized in Figure 6 shows that the decrease in the PUFA/SFA ratio is positively correlated with the TPAA levels’ drop and increased MDA levels. Concomitantly, the reduction in the PUFA/SFA ratio is positively correlated with the decrease plasma total GPx activity and inversely corralled with increase total plasma SOD activity.

#### 2.3.2. Plasma Antioxidant Trace Elements (PATE) Profile

**Figure 7:**
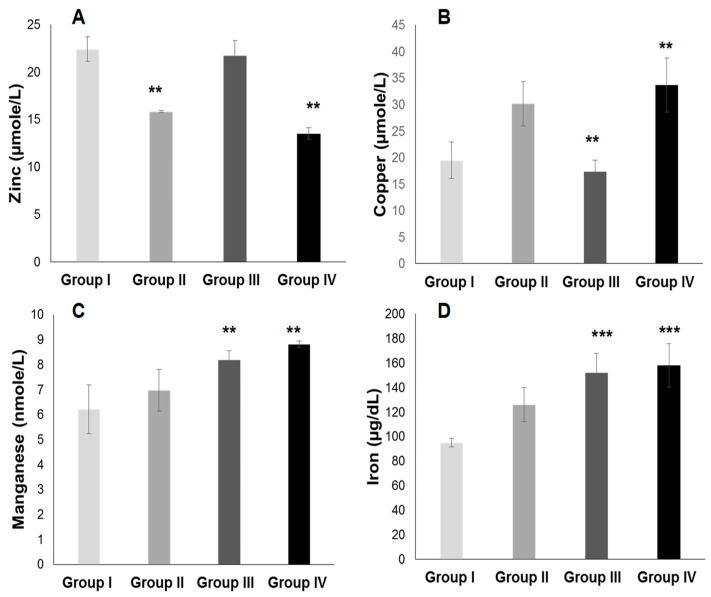
Zn (zinc), Cu (copper), Mn (manganese), and iron plasma levels in different trial groups. (**A**) Zn; (**B**) Cu; (**C**) Mn; (**D**) Iron. Group I: Healthy control participants; Group II: Diabetic participants; Group III: Hypertensive participants; Group IV: Hypertensive–diabetic participants. ** *p* < 0.01; *** *p* < 0.001.

**Figure 8:**
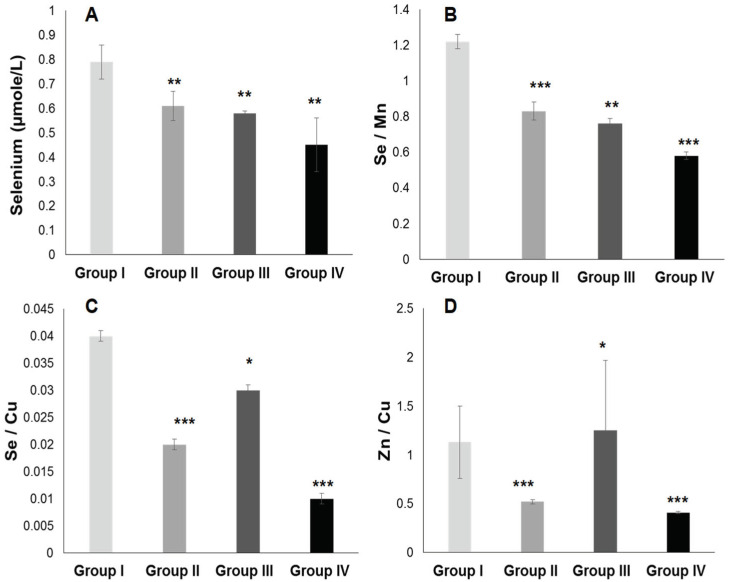
Se (Selenium) plasma levels in different trial groups. (**A**) Se; (**B**) Se/Mn ratio; (**C**) Se/Cu ratio; (**D**) Zn/Cu ratio. Group I: Healthy control participants; Group II: Diabetic participants; Group III: Hypertensive participants; Group IV: Hypertensive–diabetic participants. * *p* < 0.05; ** *p* < 0.01; *** *p* < 0.001.

**Figure 9:**
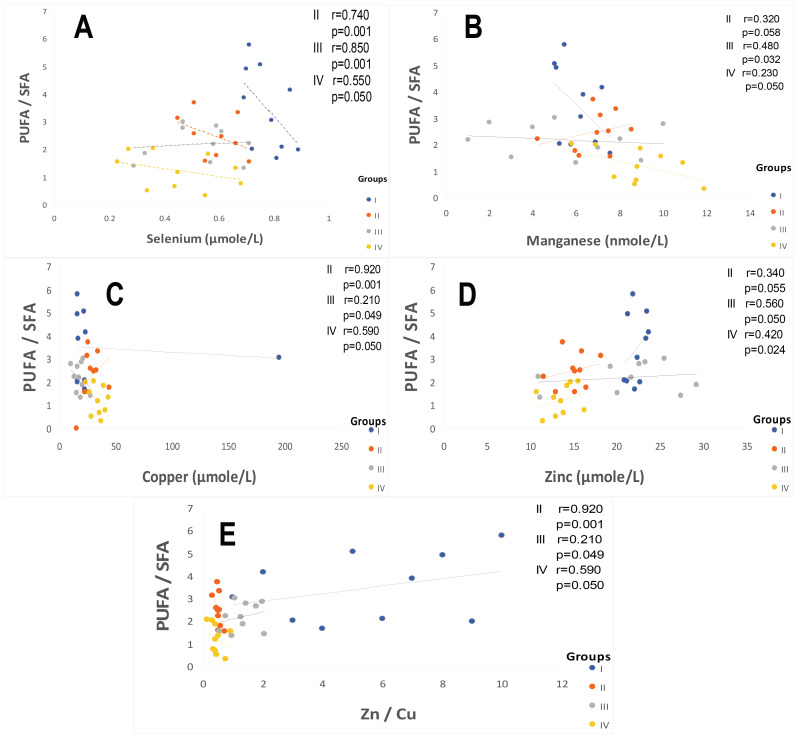
Pearson correlation between PUFA/SFA ratio and selenium (**A**), manganese (**B**), copper (**C**), zinc (**D**), and Zn/Cu ratio (**E**) plasma levels in different trial groups. Group I: Healthy control participants; Group II: Diabetic participants; Group III: Hypertensive participants; Group IV: Hypertensive–diabetic participants. PUFA: polyunsaturated fatty acids; SFA: saturated fatty acids.

Figure 7 and Figure 8 show that the PATE profile is modified during the development of systemic arterial hypertension (groups III and IV) and type 2 diabetes mellitus (group II) compared to the control group. Indeed, plasma levels of selenium (Figure 8A) and zinc (Figure 7A) gradually decrease, while plasma levels of copper (Figure 7B), manganese (Figure 7C), and iron (Figure 7D) gradually increase (*p* < 0.0001). The Se/Mn (Figure 8B), Se/Cu (Figure 8C), and Zn/Cu (Figure 8D) ratios are significantly lower in hypertensive–diabetics (group IV) compared to groups II and III (*p* < 0.0001). Pearson correlations data summarized in Figure 9 showed that the decrease in the PUFA/SFA ratio is positively associated with the reduction of Se and Zn. Conversely, the decline in the PUFA/SFA ratio is inversely associated with the increase in Cu, Mn, and Fe in hypertensive–diabetics (group IV) compared to the other groups.

## 3. Discussion

The data from this study show that the crucial relationship between plasma antioxidant altered trace elements (ATE) and plasma fatty acid ratio unbalance can be considered as a new approach to the management of systemic arterial hypertension (Groups III and IV) in diabetic subjects (Group II). Our investigation can be supported by some interconnected disorders between the ATE–FA ratio and other parameters that affect vascular endothelial dysfunction: (i) cardiometabolic syndrome, particularly lipid disorders that are linked to insulin resistance and visceral adipose tissue (VAT) accumulation; (ii) oxidative stress damage (ATE related to SOD, GPx, CAT, and GSH) and a chronic inflammatory state (Hs-CRP and ferritin); (iii) saturated/unsaturated fatty acid ratio imbalance and athero-thromboembolic risk (Hcy, Ox-LDL, ET−1, and Lp (a)). All these disorders are generated by glucolipotoxicity predisposing to heart failure, which can progress to atherosclerotic ischemia and myocardial infarction in hypertensive diabetic patients [69]. In this study, some points need to be clarified.

### 3.1. The First Major Point Is Linked to the ATE–Fatty Acids–Lipid Disorders

In our investigation, the lipid abnormalities are represented by the increase in plasma triglycerides, total cholesterol, and LDL cholesterol associated with the depletion of plasma HDL cholesterol in groups III and IV versus group control. These lipid profiles are significantly related to an increase in the non-esterified free fatty acids (NEFFA) and disturbances in the fatty acid ratio. We found a simultaneous depletion of the PUFA/SFA ratio and the % of linolenic acids (PUFA-ω3). Concurrently, selenium and zinc are decreased, while manganese, copper, and iron are increased in groups III and IV versus the control group. The mutual influences between ATE–fatty acids and metabolic syndrome cardiovascular risk factors are poorly described in hypertensive–diabetic patients. However, some mechanisms have been proposed to elucidate the interactions between LDL dyslipidemia and SFAs, particularly palmitic (C16:0) and myristic (C14:0) acids [70]. Among ATE, zinc and selenium are the minerals most correlated with lipid metabolism and cardiovascular risk factors in diabetic hypertensive subjects [71]. Indeed, zinc is an important cofactor in the activity of Δ6 desaturase, such as the hepatic stearoyl-CoA desaturase [72]. This enzyme converts stearate into monounsaturated fatty acid (oleate biosynthesis from stearate). Several studies have shown that zinc deficiency causes decreased linoleic acid (C18:2n-6) metabolism to arachidonic acid (C20:4n-6) via the intermediates, 7-linolenic acid (C18:3n-6) and dihomo−7-linolenic acid (C20:3n-6) [73,74,75].

In our investigation, we observed a decrease in the PUFA/SFA ratio simultaneously with the reduction of linolenic acid (PUFA ω-3). The PUFA/SFA ratio exhaustion is associated by a drop in zinc concentrations in group IV and III versus the control group. This depletion is concomitant with a marked increase in linoleic acid (PUFA ω-6), which may explain the rapid decrease in long-chain derivatives of EPA and DHA. If we take into account the competition between PUFA ω-6 and PUFA ω-3, this can be explained by the desaturation and elongation of the ∆−6 and ∆−5 desaturase pathways [76].

In this context, the overload in PUFAω-6 would direct the synthesis pathway toward arachidonic acid (C20: 4; ω-6). In our study, we found a strong correlation between the PUFA/SFA ratio both with zinc levels in groups IV and III compared to group II and controls. In contrast, our study did not show that plasma levels of Cu, Mn, and iron significantly change the fatty acid profile. However, the Zn/Cu molar ratio is negatively correlated with the PUFA/SFA ratio in hypertensive groups III and IV without affecting the diabetic group (group II) compared to the control group.

In addition, it is important to emphasize that the Zn/Cu molar ratio positively associated with the TG/HDL-c ratio and inversely coupled with HDL-c/LDL-c in the hypertensive groups. It is likely that the imbalance between Zn and Cu may be due to an alteration of linoleic acid (LA): gamma-linolenic acid and acide dihomo-gamma-linoléique (DGLA) ratio in dyslipidemia. Indeed, active conversion of LA to its metabolites (DGLA) depends on the trace elements levels (Zn higher and Cu lower) for increased desaturase activity [77]. Some studies have shown that the decrease in the Zn/Cu molar ratio correlated with fatty acids dyslipidemia and high blood pressure could significantly predict the risk of cardiovascular stroke [78,79].

Conversely, plasma copper levels are increased by 42% in group IV and 35% in group II, but very little in group III (hypertensive), while the % of total AGS are increased in all groups. This highlights that copper is an indicator of the diabetes evolution toward hypertension and not inversely. This observation suggests that low appropriate incorporation of copper into Cu/Zn-SOD may not be sufficient to prevent lipid peroxidation in hypertensive–diabetic subjects (group IV and II). Curiously, copper and zinc metabolism are opposed during the transition from hypertension to diabetes. In this investigation, we did not account for the consumption of trace elements in food intake, as this was not in the objectives of this study. However, some studies have described that plasma ATE levels depend closely on food intake [80]. In addition, previous work has shown that low dietary Se intake increases blood pressure [81].

### 3.2. The Second Point Is Linked between ATE–Fatty Acids–Oxidative Stress (OxS)

It should be noted the important expansion of visceral adipose tissue (VAT) leading to significant lipolysis and the release of important non-esterified fatty acids (NEFA) serum levels, which inaugurates all the disorders observed in the hypertensive–diabetic group (IV), which explains the dyslipidemia with strongly increased lipotoxicity. In this regard, lipid peroxidation plays a crucial role in the OxS onset. OxS is due to the antioxidant system failure (SOD, GPx, Se, Zn, Cu, and Mn) and the burst inflammation (Hs-CRP, fibrinogen, ferritin) in the hypertensive (groups III, IV) and diabetic groups (II). These physiological disruptions increase ROS production and circulating levels of malondialdehyde, which decrease total antioxidant protection (TAS). Our data are in agreement with many recent studies conducted in hypertensive–diabetic patients [82,83,84].

In this discussion point, our attention was focused particularly on manganese. Interestingly, we observed that plasma Mn levels are significantly increased in both hypertensive (III, IV) and diabetic (II) groups compared to the control group. These data can be explained by a competitive effect on transferrin between Mn and iron, since they are transported by this same protein. In our investigation, the iron balance in groups III and IV shows hyperferritinemia compared to the control group. This explains the mobilization of iron, which, by binding to transferrin, maintains Mn in an unbound state and leads to Mn accumulation in blood [85]. This will result in a displacement of iron from its binding sites (ferritin) on membrane phospholipids integrating PUFAs and would lead to lipid peroxidation. It is important to note that iron is constantly pro-oxidant, whereas Mn was an antioxidant. This explains that the interactions between Mn, Fe, and ferritin are intimately linked and that they can lead to dysregulation of mitochondrial levels of Fe, Mn, copper, and zinc [86].

It appears that the balance of oxidative and reducing forces is therefore subtle in the hypertensive–diabetic patient [87]. Based on our new data, we will recommend not supplementing Mn in hypertensive–diabetic subjects if they have hyperferritinemia associated with dyslipidemia. On the other hand, if iron toxicity is not proven (absence of hypersidermia), Mn is protective against polyunsaturated fatty acids peroxidation, as has been shown in positive studies [88]. In addition, Mn bioavailability lack in mitochondria induces SOD-Mn inactivity, which can aggravate OxS effects. Indeed, it is described that mitochondria are particularly vulnerable to OxS because they consume more than 90% of cellular oxygen. The production of superoxide anion is linked to an escape during the process carried out in the respiratory chain, globally proportional to its functioning [89].

Nevertheless, in this study, it seems that the activity of cytosolic SOD-Cu/Zn is not slowed down, at least not by lack of Cu and/or Zn. Similarly, in experimental models of ischemia/reperfusion, some authors have noted a specific reduction in Mn-SOD activity, without affecting CuZn-SOD activity, although an increase in the concentration of lipid peroxides at the mitochondrial level has been found to be increased [90]. It should be noted that erythrocytes, which are devoid of mitochondria, have a low Mn content in diabetic subjects [91]. Our data suggest that antioxidant defense could be preserved in the cytosol, while it would be altered in the mitochondria [92]. It is interesting to recall that Mn is involved in endothelial dysfunction via NO production, and that it was found to be elevated in the hypertensive–diabetic groups versus the control group, which may explain that Mn represents a transition trace element between vascular pathology (hypertension) and metabolic pathology (diabetes). This same observation had been noted in the evolution of diabetes toward renal dysfunction from hypertension [93].

It is essential to add that Mn bioavailability lack can explain the hyperglycemia in hypertensive subjects (group III) and can be associated with T2DM (group IV). This observation can be explained by Mn function a cofactor for some metalloenzymes (glycolysis, gluconeogenesis, Krebs cycle) can play a critical role in the glycemia regulation (pyruvate carboxylase, GTP oxaloacetate carboxylase, isocitrate dehydrogenase, malate dehydrogenase, phosphoenolpyruvate carboxykinase) [94]. In this study, special attention is given to selenium (Se). Few studies have focused on Se in hypertension associated with diabetes (group IV). In this investigation, selenium represents the link between homocysteine (hCys), oxidative stress (GPx activity), fatty acid unbalance, and inflammation (Hs-CRP) during the SAH evolution.

In our investigation, it does not appear that groups II, III, and IV are depleted in Se, because the serum Se levels are not lower than 80 µg/L, as has been described in another study [95]. The current study showed that there is a strong relationship between Se and fatty acid profile. We found a positive correlation between decreased plasma Se and concurrently falling n−3 PUFA levels, GPx activity, and GSH/GSSG ratio in groups IV and III compared to groups II and controls. Indeed, n−3 PUFAs are vulnerable to lipid peroxidation, which leads to OxS and inflammation. In agreement with our findings, Se supplementation has been described as protective against PUFA peroxidation via GPx activity [96,97]. Several studies describe that Se incorporated into selenoprotein *p* protects the oxidation of n−3 PUFAs and inflammation in cardiovascular disease [98,99,100].

It is important to emphasize that we found a positive association between Se plasma levels reduction and Se/Cu and Se/Mn depletion ratios in groups II and IV. Concomitantly, PUFA ω6/PUFA ω3 ratio and serum fibrinogen levels increase, which is an aggregating factor. We suppose that Se influences platelet function and the thromboxane/prostacyclin balance [101,102]. Furthermore, previous studies have shown that a GPx activity drop in erythrocytes leads to an accumulation of hydrogen peroxide linked to lipid peroxidation, which can cause an inhibition of SOD activity [103]. We found a positive correlation between PUFA/SFA ratio and GPx or GSH levels in these groups. Conversely, the correlation is negative between PUFA/SFA ratio and tSOD or eSOD or catalase (Figure 7).

Our data are similar to several studies [104,105,106]. It seems that antioxidant protection by TE against OxS damage in groups III and IV is important to protect endothelial cell membrane PUFAs from lipid peroxidation, which protects the vascular endothelium from atherosclerosis and thrombogenesis [107]. It has been described that Se actions are exerted through the p38 MAP kinase and NF-κB signaling pathways [108]. Furthermore, Se is able to inhibit the expression of endothelial adhesion molecules generated by the atherosclerotic process, such as VCAM−1 (vascular cell adhesion molecule−1), ICAM−1 (intercellular adhesion molecule−1), and E-selectin [109].

Recently, the importance of the link established between oxidative stress and hypertension has been highlighted through the use of experimental animal models of hypertension. Initial studies have focused on the most relevant reactive oxygen species (ROS) and nitrogen species (RNS), such as superoxide anion, hydrogen peroxide, and peroxynitrite. The hypothesis would be that ROS and RNS modify complex signaling pathways that promote or correct hypertension. Post-translational oxidative modifications by trace elements induced important protein targets of redox proteomics via redox signaling pathways. Furthermore, traces elements interact with the inflammasome activation and endoplasmic reticular stress in the development of hypertension [110].

In addition, the hypothesis would be that modification in the trace elements levels in the body are the main contributors to the development of diseases transitioning from a healthy to a pathological state. Metal trace elements and nonmetallic trace elements can help in the diagnosis of atherosclerosis [111]. In this perspective, the association between the essential trace elements mixture is beneficial to avoid the atherosclerosis risk development in a diabetic hypertensive patient [112]. Finally, new data highlight the central role of trace elements in the pathophysiology of cardiovascular diseases. Recent findings show that trace elements are able to induce changes at the epigenomic and epitranscriptomic levels. In addition, the newly discovered mechanisms could also help identify other therapeutic targets relevant for future medical applications in the prevention and treatment of hypertension [113].

### 3.3. The Third Crucial Point Is Related to the ATE–Fatty Acids–Atherothroboembolic Risk

In our study, the mutual relationship between ATE–fatty acid and atherothroboembolic biomarkers (Ox-LDL, ET−1, Hcy, and Lp(a)) are very complex. To our knowledge, no study has investigated these interactions. Taking our data together, we found a significant synergistic increase in oxidized LDL, homocysteine, endothelin, and lipoprotein (a) in hypertensive groups (III and IV).

Regarding the oxidative modified LDL (Ox-LDL) pathway, our data has highlighted that Ox-LDL has been widely accepted as a primary mediator of atherosclerosis in hypertension [114]. Ox-LDL is implicated in atheromatous plaques formation because it is dense and small in size, which allows it to infiltrate the arterial wall [115]. In this study, the relationship between Ox-LDL, ATE plasma levels, PUFA/SFA ratio, and PUFA-ω3/ω6 ratio may be relevant in determining the resistance of LDL to oxidative modification. Some studies have shown that plasma LDL-cholesterol undergoes strong oxidation in the presence of increased copper levels (the case of groups II and IV) and a high ratio of PUFA ω6/PUFA ω3 [116]. It is important to emphasize that saturated fatty acid plays a major role in the Ox-LDL formation [117]. Indeed, in our study, our attention is focused on Ox-LDL and PUFA/SFA ratio simultaneously associated with zinc depletion and copper enhancement. Concomitantly, we found a significant increased arachidonic acid, and we observed a marked amplification in % linoleic acid (PUFA-ω6). This explains the strong Ox-LDL rise and decline in long-chain derivatives of PUFA-ω3 (EPA + DHA) in group IV. It should be noted the competition between PUFA-ω3 and PUFA-ω6 via the desaturation and elongation pathways activated by ∆−6 and ∆−5 desaturases-zinc dependent [118]. The dietary zinc reduces the ∆−6 desaturases activity metabolizing linoleic acid into arachidonic acid [119]. It is highly likely in the hypertension–diabetes association, the excess in PUFA-ω6 would direct the synthesis arachidonic acid (C20:4; ω6) pathway, significantly increased in group IV compared to group II. The increase in arachidonic acid and simultaneous depletion of PUFA-ω3 and zinc may be explained by an overproduction of Ox-LDL, because PUFA-ω3 inhibits the Ox-LDL [120].

Conversely, the arachidonate enhancement can lead either to the prostaglandins production converted to thromboxane A2 (vasoconstrictor and platelet aggregator factor) via the cyclooxygenase action [121], or leukotrienes synthesis under the 5-lipoxygenase effect [122]. Furthermore, previous studies have shown in diabetic subjects with or without coronary insufficiency that arachidonic acid is incorporated more into the membrane phospholipids of blood platelets [123]. In this event, the risk of platelet aggregation increases and could lead to the development of thrombosis [124]. On other side, we found a positive association between Ox-LDL–hyperhomocysteinemia and the decrease in total PUFAs, particularly EPA and DHA in groups II, III, and IV versus the control group.

Our results are in agreement with several studies that prove that homocysteine levels influence Ox-LDL synthesis and disrupts plasma levels of eicosanoids derived from PUFAs [125,126,127]. Some studies have shown that hyperhomocysteinemia–Ox-LDL interactions stimulate the macrophage migration (Ox-LDL is a signaling pathway of immune system) in the subendothelial space of the vascular endothelium and leads to the foam cell formation that plays a crucial role in atherosclerotic lesions [128,129], which has been found in ischemic cardiopathy coronary insufficiency [130,131].

Regarding total homocysteine (tHcy) interactions in this investigation, we noticed the link between hyperhomocysteinemia, a sudden drop in EPA + DHA levels, and significantly lower plasma Zn and Se levels in group IV. Homocysteine accumulation in plasma may be due to either excessive production or its low catabolism. This result can be explained by the betaine-homocysteine methyltransferase and methionine synthase activities that have altered to convert hCys to methionine since they are dependent on zinc [132]. Zn and Se deficiency appears to be the most plausible response that demonstrates the hCys accretion in the blood. This result also explains the plasma glutathione depletion (GSH and GSH/GSSG ratio) observed in groups III and IV. Through the methylation process, hCys appears to be crucial for the metabolism of polyunsaturated fatty acids and their distribution in tissues. It seems that methyl function deficiency due to hyperhomocystenemia explains a drop in the PUFAs synthesis due to an elongation lack of the carbon chains of linolenic acid (PUFA ω3) observed in group IV [133]. It is unclear what mechanisms are underlying the correlation between hCys-fatty acid ratio-ATE in endothelial dysfunction, which deserves further study, although its involvement in atherosclerosis and thrombogenesis seems plausible as an independent factor in hypertension [134]. The limited data currently available in the literature do not allow definitive conclusions to be drawn on the relationship between Hcy and PUFA ω3.

Regarding the lipoprotein (a) pathway in this study, we showed a high association between increased Lp(a) levels and elevated PUFA ω6/PUFA ω3 ratio in diabetic (group II) and hypertensive (groups III and IV) participants. Recently, studies of dietary interventions support our data by the fact that the increase in serum Lp(a) levels is associated with a decrease in unsaturated fatty acids and an increase in saturated fatty acids [135,136]. It is interesting to note that serum Lp(a) level increases are positively correlated with EPA plasma level decreases but not DHA plasma levels. Our results are confirmed by other studies [137]. As previously argued for OxS statut, an association has been observed between increased serum Lp(a) levels and serum Ox-LDL levels. In our study, we found a positive association between elevated Lp(a) and high plasma copper levels in group IV. Our observation has been found in other studies [138]. This oxidative process leads to oxidized Lp(a) involved in atheromatous plaques development in hypertension [139]. On the other hand, it is possible that statin treatment increases serum Lp(a) levels since the hypertensive patients in this study are treated with statins, but these data remain controversial [140].

Regarding the endothelin−1 (ET−1) pathway in our investigation, we did not find an association between PUFA ω6/PUFA ω3 or PUFA/SFA ratios and serum ET-I levels; however, increased amounts of ET−1 are associated with the total SFA, particularly with palmitic acid in groups III and IV. It is likely that endothelin is strongly secreted in the presence of saturated but not unsaturated fatty acids. Our study data can be explained by the activation of protein kinase C (PKC) family signaling pathway induced by palmitic acid, which allows the ET−1 induction [141]. Previous studies have shown that PKC stimulatory effect on ET−1 gene expression has been found in brain microvascular endothelial cells [142]. The transcription factor AP−1 (activator protein−1-dependent), which plays an important role in ET−1 gene transactivation [143], is a potential target of the PKC signaling pathway [144]. Our data are validated by other studies [145]. Furthermore, we showed a strong association between increased serum ET-I levels and hyperhomocysteinemia, but not with serum Lp(a) levels in group IV. Interestingly, an interaction was found between elevated serum ET-I levels and zinc deficiency in group IV.

Several studies have shown that zinc modulates endothelin−1 signaling in vascular endothelial and smooth muscle cells [146,147,148]. In our study, the decrease in zinc levels due to deficiency or sequestration by SOD-Zn/Cu appears to increase endothelin synthesis [149], since OxS is described as a factor aggravating endothelin formation in hypertension [150].

### 3.4. The Specific Point Is Linked to Relationship between Insulin Resistance/Hypertension/Hyperinsulinism in Hypertensive Patient without Diabetes

Previous epidemiological data have observed that subjects who present high plasma insulin levels show both a disturbance in blood pressure and altered peripheral insulin sensitivity (commonly glucose intolerance) compared to healthy subjects (normoinsulinic and normotensive). Several mechanisms are proposed to explain the association between hyperinsulinemia and insulin resistance in patients with hypertension (SAH) without T2DM (group III). In the Results section, we showed that group III showed a high waist circumference (according to the NCEP-ATIII definition metabolic syndrome), which highlights abdominal adiposity. The development of visceral fat mass under the compensatory hyperinsulinism is responsible for significant fatty acids free flow release (lipolysis), which will become important energy substrates for skeletal muscle (SM) via the Randel cycle [13]. This will result in inhibition of peripheral glucose utilization, particularly by SM, and in return stimulate hepatic gluconeogenesis [151], which can explain insulin resistance, glucose intolerance, and, chronically, the hypertension development [152].

The blood pressure disturbance can be explained by the following arguments, namely altered renal sodium reabsorption via Renin–Aldosterone–Angiotensin System (RAAS) activity, increased water retention, activation of sympathetic nervous system, endothelial dysfunction, modifications in vascular remodeling (hypertrophy of vascular smooth muscle), impaired insulin-stimulated NO (nitric oxide) pathway and electrolyte imbalance (sodium, potassium, magnesium). In addition, compensatory hyperinsulinemia in hypertensive patients can activate the MAPK (mitogen-activated protein kinases) pathway, leading to increased vasoconstriction and impaired basal blood flow, particularly in the lower limbs [153,154,155,156].

### 3.5. The Particular Point Is Related to Relationship between Sex/Gender and Cardiometabolic Syndrome–Hypertension–Type 2 Diabetes Mellitus (T2DM)

Our investigation highlights that T2DM vascular complications progress rapidly to arterial hypertension, but not conversely. Indeed, several investigations [157,158] and recently confirmed by a large global epidemiological study [159] have described that sex/gender has a significant impact on cardiovascular diseases, particularly hypertension and diabetes, but also on the evolution of the disease in response to drug treatments [160]. Commonly, T2DM appears to be more common in women, while type 1 diabetes is more common in men [161]. This may be explained by the higher insulin resistance status in women than in men since adolescence [162].

Several mechanisms are proposed to explain gender/sex differences. Women appear to be more predisposed to T2DM risk factors compared to men, linked to the metabolic syndrome, with generally stronger associations in women than men [163], specifically with obesity in women as a prominent T2DM risk factor [164]. In addition, hormonal fluctuations related to reproductive function are specific to women and do not occur in men. Pregnancies may reveal pre-existing metabolic abnormalities, leading to the diagnosis of gestational diabetes and blood pressure disturbance, which appears to be the most important risk factor for progression to patent T2DM [165] and also hypertension [166]. In addition, menopause increases the cardiometabolic risk profile in women, but not in men, which explains why women with T2DM have a higher relative cardiovascular risk, particularly arterial hypertension [167] and vascular complications, such as macro-microangiopathy [168]. These data could partially explain the superiority of women relative to men in the incidence of cardiovascular disease.

### 3.6. The Important Point Is Linked to Relationship between Hs-CRP-Systemic Inflammation and Hypertension with or without Type 2 Diabetes Mellitus

In the present study, the acute-phase systemic inflammatory process is characterized by a significant increase by hs-CRP serum levels in patients with hypertension, similar to diabetic patients. Our results are in agreement with some epidemiological investigations associating CRP levels with hypertension [169,170] and type 2 hypertensive diabetes [171] as an independent risk factor for screening hypertension [172,173].

However, recent scientific knowledge about CRP as a pivotal role in the inflammatory process in various pathologies remains poorly understood and sometimes controversial [174]. Nevertheless, the majority of studies have focused on the CRP value as a predictive event of cardiovascular severity, mainly ischemic stroke and coronary heart disease [175,176,177,178]. Interestingly, we found a significant positive correlation between CRP and the triad: homocysteine (Hcy)–endothelin (ET−1)–Lp(a). This relationship is complex and poorly elucidated in the literature, but some mechanisms can be proposed to explain this association, which can be considered a predictive factor of artery atherosclerotic stroke via endothelial dysfunction and vascular stiffness [179,180].

Regarding homocysteine, singular in vivo and in vitro studies have examined the effect of Hcy on CRP expression in vascular smooth muscle cells (VSMCs) in both mRNA and protein levels. The most relevant results show that Hcy is able to initiate an inflammatory response in VSMCs by stimulating CRP production, since CRP is produced locally by VSMCs and is able to stimulate migration and proliferation of VSMCs [181], which affects vascular endothelial function. These results provide new evidence for the link between CRP and Hcy in the atherosclerosis pathogenesis, which is mediated by the NMDAr-ROS-MAPK (N-methyl-D aspartate receptor-reactive oxygen species-mitogen-activated protein kinase) signaling pathway [182] independently of the proinflammatory cytokines effects.

Regarding endothelin, the studies are controversial whether or not it is influenced by elevations in CRP [183,184]. However, in patients with acute ischemic stroke, a positive correlation between CRP and endothelin−1 was reported [185]. The concomitant increase in endothelin−1 and CRP most likely reflects ischemic lesions post-stroke in order to adapt to the therapy.

Regarding Lp(a), few studies have explored the relationship between CRP and Lp(a). However, currently Lp(a) has regained renewed interest with intense exploration, particularly in the therapeutic field [186,187,188]. In arterial hypertension, the increase in Lp(a) levels is explained by renal endothelial dysfunction associated with oxidative stress. Indeed, Lp(a) is vulnerable to oxidation and leads to the development of atheromatous plaques [189]. In addition, the resurgence of Lp(a) in hypertensive subjects could also be related to drug treatment where Lp(a) is very little reduced by statins [190]. It is interesting to note that some studies have shown that Lp(a) only increases significantly when CRP levels are high. This highlights a synergistic effect of Lp(a) and CRP on cardiovascular risk death in patients with acute myocardial infarction [191].

### 3.7. The Prominent Point Is Related to Potential Mechanism and Clinical Implications of SOD Activity in Diabetic and Hypertensive Patients with or without Type 2 Diabetes Mellitus

In our study, we found a significant increase in plasma SOD1 and erythrocyte SOD2 levels proportionally with a rise in MDA in patients with T2DM or hypertension or their combination versus the healthy group. This indicates that excessive production of superoxide anions (O2^.^) linked to lipotoxicity (hyperglycemia and dyslipidemia) strongly activates SOD, which produces significant amounts of H_2_O_2_ (hydrogen peroxide) that cannot be completely eliminated by catalase and GPx, which explains their drop activities. Also, this indicates that oxidative stress damage could be explained by different cellular responses to endothelial dysfunction and Langerhans islets beta cells dysfunction. Some potential mechanisms may explain these results and may point to clinical implications.

A previous study has shown that a high hydrogen peroxide level due to superoxide anion overproduction inhibits catalase activity [192]. H_2_O_2_ has been shown to be a potent activator of the NF-ƙB signaling pathway, which is involved in the gene activation of proinflammatory cytokines [193,194] and which can induce CRP synthesis [195].

Some preceding studies indicate that superoxide anion plays an important role in increasing blood pressure by activating the renin–angiotensin–aldosterone system [196,197], which leads to overproduction of angiotensin II (hypertensive hormone). Interestingly, the SOD activity preferentially uses zinc as a cofactor to eliminate superoxide anion and not copper, which explains the zinc depletion in our study. By decreasing superoxide anion levels, the actions of SOD are reflected in the decreased angiotensin II, but also increased vascular NO levels, playing an important role in vasodilatation [198,199]. It has been suggested that the increase in plasma SOD concentration associated with diabetic and hypertensive patients may reflect decreased the enzyme binding in the beta cells [200] and vascular endothelial smooth muscle cells [201]. Some meta-analysis data prove that SOD can be considered a therapeutic target with clinical application [202,203].

## 4. Materials and Methods

It is necessary to note that the cohort of this study is the same as we used in a previous investigation in hypertensive versus diabetic subjects, which confirms the similarity of participants at enrollments [8].

### 4.1. Informed Consent Statement and Ethical Considerations

This clinical study protocol (Algiers essential arterial hypertension Study) was approved by the Ethics Committee of Algerian Ministry of Public Health (ECAMPH) and conformed to the principles outlined in the Declaration of Helsinki (http://www.wma.net, accessed on 19 October 2013). Ethical approval code: The permits and ethical rules have been achieved according to the Executive Decree no. 10–90 (10 March 2010) completing the Executive Decree no. 04–82 (18 March 2004) of the Algerian Government, establishing the terms and approval modalities. An informed consent form was signed by each participant.

### 4.2. Participants and Clinical Protocol Design

**Figure 10:**
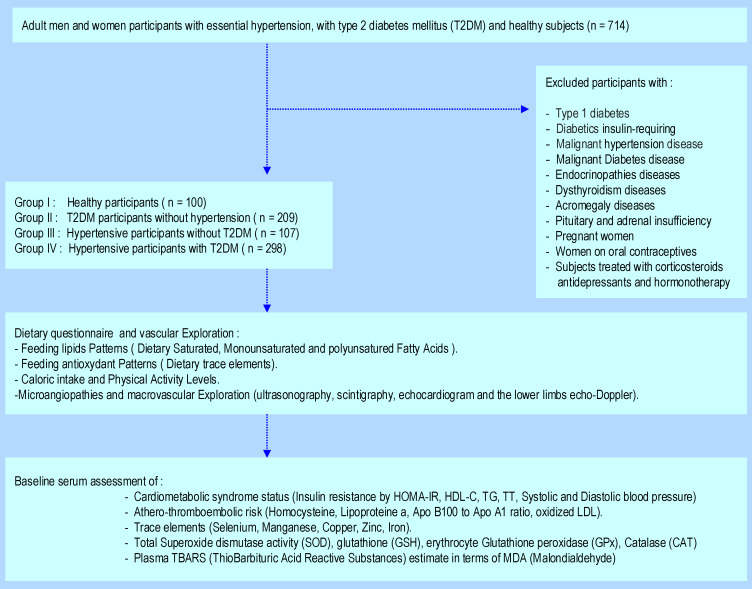
The clinical protocol design in Diabetic, Hypertensive, and Hypertensive–diabetic participants compared to Healthy participants (control group). This clinical investigation was a randomized, multicenter cross-sectional and observational design study; case-control was carried between September 2020 and October 2023. The sample size was estimated using Cochran’s formula. The all participants cohort was classified according to age and sex, with a sex ratio of men/women = 0.94. Diabetic participants were treated with metformin 300 mg/24 h, associated with sulfonylurea. Group IV was treated with a variable combination therapy: beta-blocker, calcium channel blocker, inhibitor of the angiotensin converting enzyme, and diuretic.

This clinical investigation was a randomized, multicenter cross-sectional and observational design study. Case-control was carried out between September 2020 and October 2023. All participants were admitted to the diabetology unit, Mohamed Seghir Nekkache Hospital, and diabetology-cardiology unit, Bab El Oued University Hospital Center (UHC), Mohamed Lamine Debaghine (MLD) of Algiers, Algeria. All the study parameters’ measurements were evaluated in the Biochemistry and Genetics Laboratory, UHC-MLD of Algiers. We included in the study 714 adult participants, aged between 36 and 54 years, including 397 men (M) and 317 women (F). The sample size was estimated using Cochran’s formula. The all participants cohort was classified according to age and sex, with a sex ratio of men/women = 0.94. This clinical investigation was undertaken in (Figure 10):

209 Type−2 diabetes mellitus (T2DM) participants without hypertension (Group II)

107 Hypertensive participants without T2DM (Group III)

298 T2DM participants with Hypertension (Group IV)

100 Healthy participants (Group I), without pathologies and non-smokers

Diabetic participants were treated with metformin 300 mg/24 h, associated with sulfonylurea. Group IV was treated with a variable combination therapy: beta-blocker, calcium channel blocker, inhibitor of the angiotensin converting enzyme, and diuretic. The drug doses were stable throughout the study. Diabetes age and the presence of hypertension in Group IV were variable, between 5 and 10 years. In this study, we excluded all subjects with endocrinopathies, such as Cushing’s disease, dysthyroidism, acromegaly, pheochromocytoma, pituitary, and adrenal insufficiency. We also excluded pregnant women and those on oral contraceptives. Similarly, patients treated with corticosteroids, antidepressants, hormone therapy, and type 1 diabetics were excluded. No participants were insulin-requiring. In the day hospital, we explored microangiopathies and macrovascular complications by ultrasonography, scintigraphy, echocardiogram, and the lower limbs echo-doppler. The participants benefited from the supra-aortic trunks Doppler to calculate the intima-media diameter. All clinical explorations participants have been examined by the same physician.

### 4.3. Cardiometabolic Syndrome (CMS) Screening

CMS was confirmed according to the definition of the NCEP/ATPIII (National cholesterol education program third adult treatment panel/Adult Treatment Panel III) criteria [204]. The CMS was identified by the presence of three or more disorders of CMS clusters as follows: (1) visceral obesity; (2) high plasma triglyceride level; (3) low plasma HDL cholesterol level; (4) high fasting plasma glucose; (5) a blood pressure disturbance. Insulin resistance was calculated by the homeostasis model assessment insulin resistance (HOMA-IR) method: HOMA index = fasting glucose (mmol/L) × fasting insulin (mU/L)/22.5 [205]. The percentage of body fat (BF) was calculated using the formula: (1.2 × BMI) + (0.23 × age) − (10.8 × S)–5.4 (S is the gender correction factor) [206]. The SBP (systolic blood pressure) and DBP (diastolic blood pressure) were measured in the prone position of the two arms, three times and two minutes after ten minutes of rest using a validated Omron 705 CP type BP monitor (Omron Healthcare Europe BV, Amsterdam, The Netherlands) [207]. Hypertension was measured via blood pressure defined based on the WHO (World Health Organization) standard definition as SBP of ≥140 mm Hg and/or DBP of ≥90 mm Hg and/or currently taking antihypertensive medications [208].

### 4.4. Classification and Diagnosis Criteria of Hypertension

In our study, SAH was diagnosed and confirmed according the recommendations of European Society of Hypertension (ESH) criteria [209]. Recently, these criteria have been slightly modified [210]. According to the main ESH guidelines, it is recommended to diagnose hypertension when a subject’s systolic blood pressure (SBP) in a medical consultation is ≥140 mm Hg and/or diastolic blood pressure (DBP) is ≥90 mm Hg after repeat measurements in the medical office, ambulatory, and at home. These criteria apply to all adults (>18 years) so that the therapeutic targets approaches are the same for all hypertension categories. The classification of hypertension grades according ESH as follows:

Optimal: <120 SBP and <80 DBP

Normal: 120–129 SBP and 80–84 DBP

High-normal: 130–139 SBP and/or 85–89 DBP

Grade 1 hypertension: 140–159 SBP and/or 90–99 DBP

Grade 2 hypertension: 160–179 SBP and/or 100–109 DBP

Grade 3 hypertension: ≥180 SBP and/or ≥110 DBP

Isolated systolic hypertension: ≥140 SBP and <90 DBP

Isolated diastolic hypertension: <140 SBP and ≥90 DBP

### 4.5. Classification and Diagnosis Criteria of Diabetes Mellitus

In this study, T2DM was diagnosed and confirmed according to the International Diabetes Federation (IDF) [211]. Diabetes can be diagnosed either by the hemoglobin A1C criteria or plasma glucose concentration (fasting or 2 h plasma glucose). The classification of T2DM according IDF as follows:

Fasting Plasma Glucose (FPG): A blood sample is taken after an 8 h overnight fast. As per ADA, fasting plasma glucose (FPG) level of more than 126 mg/dL (7.0 mm/L) is consistent with the diagnosis.

Two-Hour Oral Glucose Tolerance Test (OGTT): In this test, the plasma glucose level is measured before and 2 h after the ingestion of 75 gm of glucose. T2DM is diagnosed if the plasma glucose (PG) level in the 2 h sample is more than 200 mg/dL (11.1 mmol/L). It is also a standard test but is inconvenient and more costly than FPG and has major variability issues. Patients need to consume a diet with at least 150 g per day of carbohydrates for 3 to 5 days and not take any medications that can impact glucose tolerance, such as steroids and thiazide diuretics.

Glycated Hemoglobin (Hb) A1C: This test gives an average of blood glucose over the last 2 to 3 months. Patients with Hb A1C greater than 6.5% (48 mmol/mol) are diagnosed as having T2DM. Hb A1C remains the gold standard in identifying unrecognized diabetes mellitus and impaired glucose tolerance in hypertensive subjects.

If the diabetes cardinal symptoms are found in a subject (polyuria, polydipsia, hyperphagia, sudden weight loss), a random plasma glucose level (at any time of the day) greater than 200 mg/dL is sufficient evidence to make the diagnosis of T2DM.

### 4.6. Plasma Samples and Biochemical Analysis

The participants were admitted to the hospital at 7 am after 12 h of fasting before they consumed their drugs (therapeutic treatment). Blood samples were centrifuged at 3000 rpm for 10 min, and plasma was obtained. Fasting plasma samples were immediately put on ice and kept frozen at –80 °C until analyses were performed. Fasting plasma glucose, triglycerides (TG), total cholesterol (TC), high-density lipoprotein cholesterol (HDL-C), transaminases (ALT, AST), GGT, creatinine, and uric acid were determined by enzymatic methods using an automatic biochemical analyzer (Cobas Integra 400^®^ analyzer, Roche Diagnostics, Meylan, France). Plasma glycosylated hemoglobin (HbA1C) and microalbuminuria were determined by turbidimetry (Roche Diagnostic Systems, Basel, Switzerland). The low-density lipoprotein cholesterol (LDL-C) was calculated using Friedewald’s formula [LDL-C (mg/dL) = TC–HDL-C–TG/5.0] applied to subjects with IRS [212]. The criterion for detecting low-grade inflammation has been determined by plasma high-sensitive C-reactive protein (Hs-CRP) level and ferritin assessed using immunoturbidimetric methods on chemical Synchron analyzer LX^®^20 PRO (SYNCHRON LX^®^20 PRO; Beckman-Coulter, Inc., Fremont, CA, USA). The fibrinogen was evaluated by the chronometric Von Clauss methods using hemostasis analyzer ACL TOPTM (Biolabo, Maizy, France). Insulin concentrations were determined by RIA (RadioImmunoAssay) using commercially available kits (Human insulin specific RIA kit, EMD Millipore Corporation St. Louis, MO 63,103, USA). Apolipoprotein A1 (Apo A1), Apolipoprotein B100 (Apo B100), and Lp (a) lipoprotein were determined by Synchron LX^®^20 PRO analyzer. Homocysteinemia (Hcy) was assessed using FPIA (fluorescence polarization immuno assay) on Immulite 2000 analyzer Ref: L2KH02. The plasma oxidized low-density lipoprotein (Ox-LDL) levels were assayed according to the ELISA method previously described using OxiSelectTM LDLox Elisa Kits [213]. Endothelin−1 (ET−1) was quantified using commercially available ELISA kits (Morinaga and R&D System). Standards, reagents, and test samples were prepared and analyzed according to the manufacturer’s instructions.

### 4.7. Plasma Fatty Acids Extraction and Assay

The blood samples were taken on sodium oxalate. The total plasma lipids were separated by the Folch [214] and Dole [215] methods from 0.5 mL of plasma by adding 5 mL of a chloroform/methanol mixture (2:1 *v*/*v*) and 1 mL of 5% butyl–hydroxy–toluene in methanol. The homogenate was purified on degreased filter paper (Durieux brand without ash N°114–110 m/m). After the extraction in the heptane phase, the dry residue containing the fatty acids was taken up in 50 µL of hexane, 1 µL of the solution obtained was injected into a stationary phase capillary column of polyethylene glycol (HP-Innowax type), 30 m length, 0.32 mm inside diameter, and 0.5 µm film thickness. The assessment of saturated fatty acids (SFA), monounsaturated fatty acids (MUFA), polyunsaturated fatty acids (PUFA), eicosapentaenoic acid (EPA), and docosahexaenoic acid (DHA) were analyzed by gas chromatography on HP5890A (Hewlett-packard-normalk analyzer) series II equipped with a flame ionization detector. The carrier gas was nitrogen with a flow rate of 1 mL/min. The injector, detector, and column temperatures were 220 °C, 275 °C, and 180 °C, respectively. Adding internal standard or internal controls to samples allows the quantification of fatty acids within the sample by calculations using the area of known quantity of the internal standard peak relative to the area of the peak fatty acids. Internal standards were dissolved in 1 mL/mg of dry chloroform: methanol (2:1, *v*/*v*) containing butylated hydroxytoluene (BHT; 50 mg/L) as an antioxidant. The loss in the total amount of fatty acids extraction by our method is estimated between 5 and 10%. The NEFFA were extracted by the Duncombe method [216] and determined by microfluorimetry using a KONTRON analyzer, Power Supply SFM23, Augsburg, Germany.

### 4.8. Trace Elements Determination and Assessment Methods

Plasma trace elements (selenium, manganese, copper, and zinc) were determined by the Flame Atomic Absorption Spectrometry (Flame-AAS) technique. This method was widely employed for elements determination [217]. Trace metal determinations were performed according to protocol method previously described [218] with a flameless atomic absorption spectrophotometer (PerkinElmerAnalyst 800^®^, Burladingen, Germany). Argon was used as the purging gas. One thousand µg mL^−1^ standard solutions of zinc, copper, manganese, and selenium were used to prepare the standard curves (Wako Pure Chemical Industries, Osaka, Japan).

Zinc: A Shimadzu hollow-cathode zinc lamp was used as the source of a current of 10 mA. The spectrometer was operated at 213.8 nm in the peak height mode with 1.1 nm slit width. Plasma was prepared by dilution with deionized distilled water, plasma in a dilution of 1/100. Zinc concentrations were calculated by linear regression lines.

Copper: A Shimadzu hollow-cathode copper lamp was used as the source of a current of 10 mA. The spectrometer was operated at 324.8 nm in the peak height mode with 2.2 nm slit width. Plasma was diluted 1/10 with 0.1 N nitric acid. The standard addition method was used.

Manganese: A Shimadzu hollow-cathode manganese lamp was used as the source of a current of 10 mA. The spectrometer was operated at 279.5 nm in the peak height mode with 1.1 nm slit width. The manganese concentration was calculated by the standard addition method.

Selenium: A Shimadzu hollow-cathode selenium lamp was used as the source of a current of 10 mA. The spectrometer was operated at 196.0 nm in the peak height mode with 2.2 nm slit width. Plasma samples were diluted (1/2) with 1.0% (*w*/*v*) nickel−0.l N nitric acid solution. The standard addition method was used. Duplicate measurements were made with each sample. All glassware was tested for contamination. The measurement of all trace elements was performed by the following heating program: drying by ramp mode from 20 to 200 °C with temperature increases of l °C/s, ashing at 60 A (500 °C) for 30 s, and atomization at 210 A (2000 °C) for 5 s. We also used Randox kits (Randox Laboratories, Crumlin, UK) to confirm the results obtained by biochemical methods [219].

Iron: The determination of iron in the serum was carried out by colorimetric method.

### 4.9. Plasma Oxidative Stress Biomarkers and Analytical Process

#### 4.9.1. Total Blood Antioxidant Status (TAS), Plasma Thiobarbituric Acid Reactive Substances (TBARS), and Plasma Malondialdehyde (MDA) Levels Quantification

The TAS was analyzed by the method based on a test that measures the capacity of the biological fluids to inhibit the production of TBARS from sodium benzoate under the influence of the free oxygen radicals derived from Fenton’s reaction [220]. Among the end products formed during the peroxidation of polyunsaturated fatty acids mediated by free radicals, we assessed the plasma MDA and TBARS levels. These two biomarkers do not have the same oxidative stress specificity. However, it is the most frequently measured biomarkers lipid peroxidation [221] by MDA that is the prototype of the TBARS. The plasma TBARS were estimated according to the method described previously [222].

#### 4.9.2. SOD, GPx, Catalase Activities, and Glutathione Levels Determination

The superoxide dismutase activity (SOD) activity was measured both in plasma (total SOD) and in erythrocytes according to the method described previously [223]. The glutathione peroxidase (GPx) activity was determined in erythrocytes according to the method described previously [224]. The glutathione (GSH) levels were analyzed by spectrophotometer methods [225]. The results of erythrocyte SOD and erythrocyte GPx were expressed in U/g Hb (unit/gr. of hemoglobin). The Hb was read at 541 nm in a spectrophotometer (model 181 UV-vis, Hitachi, Ltd., Tokyo, Japan).

### 4.10. Atherothromboembolic Risk Assessment

The lipoprotein (a), homocysteine (hCys), Apo B100/Apo A1 ratio, and ox-LDL have been used as atherothrombogenic biomarkers. The measurement methods have been described previously.

### 4.11. Statistical Analysis

Considering our investigation was a randomized cohort, all data are measured normally distribution series. Results are presented as mean ± standard deviation (SD). All statistical analyses were performed with Epi-info version 5 and Statview version 5 (Abacus Concepts, Berkeley, USA). Student’s *t*-test and one-way ANOVA were used for the comparison both between the 3 groups (II, III and IV) and with the control participants (group I). A *p* value less than 0.05 was considered statistically significant. Both methods are parametric and assume normality of the data and equality of variances across comparison groups. Pearson’s coefficient (r) correlation analysis was performed to quantify associations between the plasma PUFA/SFA-PUFA-n3/PUFA-n6 ratios, trace elements profile (Se, Zn, Cu, Mn, Iron), CMS clusters, oxidative stress biomarkers (TAS, SOD, GPx, CAT, GSH, GSSG), and the atherothrombogenic risk characterized by the levels of Ox- LDL, homocysteine, and Lp(a). The results were considered significant if *p* < 0.05 (*), very significant if *p* < 0.01 (**), or highly significant if *p* < 0.001 (***).

## 5. Conclusions

The lipotoxicity represents the pivotal pathophysiological factor links for both the fatty acids unbalance ratio and altered plasma trace element status (see graphical abstract) in diabetic and hypertensive patient. The significant expansion of VAT leading to significant lipolysis and release of significant NEFA is observed in the hypertensive–diabetic group. We found a decrease in the PUFA/SFA ratio simultaneously with the reduction in linolenic acid (PUFA ω-3). Concomitantly, selenium and zinc are decreased, while manganese, copper, and iron are increased in the hypertensive and hypertensive–diabetic groups compared to the healthy group. The Zn/Cu molar ratio is positively associated with the TG/HDL-c ratio and inversely coupled with HDL-c/LDL-c in the hypertensive groups. This highlights that copper is an indicator of the evolution from diabetes to hypertension and not the other way around. We observed that plasma Mn levels are significantly increased in both hypertensive and diabetic groups compared to the healthy group. In this study, it seems that the activity of cytosolic SOD-Cu/Zn is not slowed down, at least not by lack of Cu and/or Zn. We found a positive correlation between decreased plasma Se and concurrently falling n−3 PUFA levels, GPx activity, and GSH/GSSG ratio in the hypertensive–diabetic group and the hypertensive group compared to the diabetic and healthy controls groups. We found a positive association between Se plasma levels reduction and Se/Cu and Se/Mn depletion ratios in the diabetic group and hypertensive–diabetic group. In our study, Ox-LDL and PUFA/SFA ratio simultaneously associated with zinc depletion and copper enhance. We noticed the link between hyperhomocysteinemia, a sudden drop in EPA + DHA levels, and significantly lower plasma Zn and Se levels in the hypertensive–diabetic group. We showed a high association between increased Lp(a) levels and elevated PUFA ω6/PUFA ω3 ratio in diabetic and hypertensive participants. In our investigation, we did not find an association between PUFA ω6/PUFA ω3 or PUFA/SFA ratios and serum ET-I levels; however, increased amounts of ET−1 are associated with the total SFA, particularly with palmitic acid in hypertensive–diabetic and hypertensive patients. Furthermore, we showed a strong association between increased serum ET-I levels and hyperhomocysteinemia, but not with serum Lp(a) levels in the hypertensive–diabetic group. Interestingly, an interaction was found between elevated serum ET-I levels and zinc deficiency in the hypertensive–diabetic group.

## 6. Limitations of This Study

This study is limited mainly by a supplementation investigation by trace elements and PUFA. In order to highlight the specific role of each nutrient in relation to the parameters studied. It was also necessary to take into consideration the stage of hypertension and diabetes, to confirm the effectiveness of supplementation on a clinical application. In addition, we are convinced that our study has some limitations; particularly, some medications, including oral antidiabetics (metformin); angiotensin converting enzyme (ACE) inhibitors; antiplatelets; and statins can affect plasma ATE and fatty acid levels.

## Figures and Tables

**Table 1:** Participants’ cohort characterization according anthropometric status in various clinical trial groups.

P/G	Group I	Group II	Group III	Group IV
	(N = 100)	(N = 209)	(N = 107)	(N = 298)
Age (year)	46 ± 2	50 ± 4	53 ± 5	55 ± 3
Sex-gender repartition (%)	50 ^(M)^ 50 ^(F)^	47 ^(M)^ 53 ^(F)^ ***	71 ^(M)^ 29 ^(F)^ ***	35.5 ^(M)^ 64.5 ^(F)^ ***
Body Weight (Kg)	69 ± 3	81 ± 5	79 ± 6	85 ± 2
BMI (Kg/m^2^)	23 ± 2	34 ± 4 ***	31 ± 1 ***	35 ± 3 ***
WC (cm)	76 ± 3 ^(F)^	110 ± 5 ^(F)^ ***	109 ± 4 ^(F)^ ***	113 ± 5 ^(F)^ ***
	74 ± 2 ^(M)^	94 ± 2 ^(M)^	101 ± 5 ^(M)^ ***	111 ± 3 ^(M)^ ***
WC/WH ratio	0.81 ± 0.02 ^(F)^	1.07 ± 0.01 ^(F)^ ***	0.99 ± 0.02 ^(F)^ ***	1.13 ± 0.03 ^(F)^ ***
	0.87 ± 0.02 ^(M)^	1.05 ± 0.03 ^(M)^	0.93 ± 0.01 ^(M)^ ***	1.09 ± 0.04 ^(M)^ ***
BF (%)	13.0 ± 2.55 ^(F)^	47.8 ± 5.11 ^(F)^ ***	33.7 ± 2.58 ^(F)^ ***	61.9 ± 5.22 ^(F)^ ***
	9.11 ± 0.56 ^(M)^	43.4 ± 7.13 ^(M)^ ***	31.8 ± 3.23 ^(M)^ ***	49.9 ± 5.18 ^(M)^ ***

P: parameters; G: group; Group I: Healthy control participants; Group II: Diabetic participants; Group III: Hypertensive participants; Group IV: Hypertensive–diabetic participants; M: male; F: female; N: total number of participants; BMI: body mass index; WC: waist circumference; WH: waist hips; BF: body fat percentage. The mean values are assigned from the standard error to the mean (X ± SD). The degree of significance is calculated for a risk of error α = 5%. The comparison of means is established both between the groups II, III, and IV versus control group. *** *p* < 0.001.

**Table 2:** Participants’ cohort classification according to cardiometabolic syndrome in various clinical trial groups.

P/G	Group I	Group II	Group III	Group IV
	(N = 100)	(N =209)	(N = 107)	(N = 298)
Glycemia (mmol/L)	4.62 ± 0.22	7.99 ± 0.59 **	5.33 ± 0.81 ***	9.63 ± 0.66 ***
Insulinemia (pmol/L)	67 ± 1.44	149 ± 5.25 **	126 ± 3.19 ***	174 ± 8.33
HOMA-IR	1.68 ± 0.05	4.91 ± 0.23 ***	3.68 ± 0.54 ***	7.97 ± 0.81 ***
HbA1_C_ (%)	5.11 ± 0.22	6.43 ± 0.52 **	5.64 ± 0.17 ***	9.21 ± 0.64 ***
Triglycerides (mmol/L)	1.19 ± 0.21	1.98 ± 0.32 ***	1.87 ± 0.54 ***	2.08 ± 0.62 ***
Total Cholesterol (mmol/L)	3.25 ± 0.17	5.27 ± 0.63 ***	5.89 ± 0.81 ***	6.67 ± 0.22 ***
HDL-C (mmol/L)	1.52 ± 0.22 ^(F)^	1.08 ± 0.31 ^(F)^	1.10 ± 0.47 ^(F)^ *	1.04 ± 0.19 ^(F)^ *
	1.24 ± 0.1 ^(M)^	1.06 ± 0.2 ^(M)^	1.09 ± 0.1 ^(M)^ *	0.98 ± 0.1 ^(M)^ *
LDL-C (mmol/L)	2.45 ± 0.5	3.48 ± 0.6 **	4.23 ± 0.5 ***	4.69 ± 0.3 ***
AST (IU/L)	20.2 ± 1.55	21.6 ± 4.07	23.2 ± 5.66	25.1 ± 3.27
ALT (IU/L)	20.1 ± 2.64	25.4 ± 3.07	28.2 ± 4.46 ***	34.7 ± 8.09 ***
AST/ALT Ratio	1.04 ± 0.06	0.85 ± 0.02	0.82 ± 0.01 ***	0.72 ± 0.04 ***
GGT (IU/L)	19.2 ± 7.11	28.3 ± 6.19 ***	37.4 ± 5.51 ***	62.1 ± 9.04 ***
Hs-CRP (mg/L)	2.81 ± 1.79	5.66 ± 0.91 **	5.41 ± 0.66 ***	7.82 ± 0.53 ***
Fibrinogen (mg/L)	2.97 ± 0.41	3.38 ± 0.13	3.19 ± 0.11	3.42 ± 0.10
Ferritin (ng/mL)	69.4 ± 9.22	109 ± 11.9 ***	146 ± 19.8 ***	166 ± 12.7 ***
Creatinine (µmol/L)	68 ± 3.41	71 ± 2.52	80 ± 4.11 ***	81 ± 6.32 ***
Uric acid (µmol/L)	284 ± 12	404 ± 16	321 ± 14	517 ± 18 ***
Microalbuminuria (mg/24 h)	14.3 ± 2.74	27.8 ± 4.63 ***	34.6 ± 5.27 ***	47.9 ± 6.47 ***

P: parameters; G: group; Group I: Healthy control participants; Group II: Diabetic participants without hypertension; Group III: Hypertensive participants without DT2; Group IV: Hypertensive–diabetic participants; M: male; F: female; DT2: type 2 diabetes; HOMA: Homeostasis Model Assessment; C: cholesterol; HDL: high-density lipoprotein; LDL: low-density lipoprotein; Hs-CRP: high sensitive C reactive protein; AST: aspartate aminotransferase; ALT: alanine aminotransferase; GGT: gamma-glutamyl transferase. The comparison of means is established between groups II, III, and IV versus control group. * *p* < 0.05; ** *p* < 0.01; *** *p* < 0.001.

**Table 3:** Participants’ cohort screening according cardiovascular and athero-thrombogenic profile in various clinical trial groups.

P/G	Group I	Group II	Group III	Group IV
	(N = 100)	(N = 209)	(N = 107)	(N = 298)
SBP (mm Hg)	120 ± 7	128 ± 5	146 ± 3 ***	158 ± 3 ***
DBP (mm Hg)	63 ± 4	71 ± 3	91 ± 5 ***	97 ± 6 ***
HDL-c/LDL-c	0.58 ± 0.02	0.33 ± 0.01 **	0.37 ± 0.03 ***	0.25 ± 0.02 ***
TG/HDL-c	0.87 ± 0.02	1.68 ± 0.04 **	1.70 ± 0.01 ***	1.97 ± 0.03 ***
ApoA_1_ (g/L)	1.71 ± 0.01	1.37 ± 0.05	0.85 ± 0.01 ***	0.80 ± 0.02 ***
ApoB_100_ (g/L)	0.84 ± 0.02	0.93 ± 0.05	0.92 ± 0.01	0.99 ± 0.07
ApoB_100_/ApoA_1_	0.49 ± 0.01	0.67 ± 0.01	1.08 ± 0.02 ***	1.23 ± 0.06 ***
Lp(a) (g/L)	0.21 ± 0.06	0.23 ± 0.02 **	0.34 ± 0.03 ***	0.85 ± 0.01 ***
tHcy (µmol/L)	10.3 ± 0.77	10.9 ± 0.06	15.7 ± 0.22 ***	17.4 ± 0.11 ***
Ox-LDL (µmol/L)	15.7 ± 1.08	33.6 ± 6.23 ***	47.9 ± 5.56 ***	55.3 ± 6.11 ***
ET-1 (pg/mL)	51.5 ± 8.75	77.30 ± 9.03 **	99.3 ± 5.11 ***	114 ± 7.22 ***

** *p* < 0.01; *** *p* < 0.001.

**Table 4:** Participants’ cohort screening according plasma fatty acids profile in various clinical trial groups.

P/G	Group I	Group II	Group III	Group IV
	(N = 100)	(N = 209)	(N = 107)	(N = 298)
NEFFA (µmol/L)	540 ± 25	627 ± 13 **	594 ± 77 ***	894 ± 89 ***
Total SFA (mol %)	16.2 ± 4.63	19.4 ± 3.24 **	21.7 ± 4.89 ***	34.3 ± 5.29 ***
Lauric acid	0.37 ± 0.03	0.48 ± 0.06 *	0.50 ± 0.03 **	0.61 ± 0.03 **
Myristic acid	0.40 ± 0.07	0.68 ± 0.02 *	0.76 ± 0.04 ***	0.98 ± 0.04 ***
Palmitic acid	11.01 ± 1.02	15.1 ± 1.05 **	16.5 ± 1.01 ***	25.1 ± 1.07 ***
Stearic acid	4.44 ± 0.51	3.17 ± 0.11	3.99 ± 2.81	7.61 ± 1.78 **
Total MUFA (mol %)	17.2 ± 1.49	21.7 ± 1.98	23.9 ± 1.33 ***	29.7 ± 1.71 ***
Total PUFA (mol %)	49.6 ± 3.23	47.9 ± 2.88	47.6 ± 3.21	40.2 ± 3.55 **
Linoleic acid(w6)	40.2 ± 1.66	37.1 ± 1.99 *	36.1 ± 2.55 *	27.9 ± 1.50 ***
Linolenic acid (w3)	1.31 ± 0.13	0.71 ± 0.20	0.73 ± 0.32 ***	0.41 ± 0.13 ***
Arachidonic acid	8.1 ± 1.44	10.1 ± 0.87 **	10.8 ± 1.94 ***	11.9 ± 1.94 ***
PUFA/SFA	3.06 ± 0.69	2.46 ± 0.88	2.19 ± 0.65 ***	1.17 ± 0.67 ***
PUFA w6/PUFA w3	30.6 ± 12.2	52.2 ± 9.95 ***	49.4 ± 7.96 ***	87.5 ± 11.5 ***
EPA (mol %)	2.98 ± 0.33	1.81 ± 0.22 ***	1.75 ± 0.21 ***	1.22 ± 0.17 ***
DHA (mol %)	2.55 ± 0.61	2.08 ± 0.89 ^ns^	1.99 ± 0.11 ***	0.88 ± 0.03 ***
EPA+DHA (mol %)	5.53 ± 0.94	3.89 ± 1.11 *	3.74 ± 0.32 ***	2.10 ± 0.20 ***

P: parameters; G: group; Group I: Healthy control participants; Group II: Diabetic participants; Group III: Hypertensive participants; Group IV: Hypertensive–diabetic participants; NEFFA: non-esterified free fatty acids; SFA: saturated fatty acids; MUFA: monounsaturated fatty acids; PUFA: polyunsaturated fatty acids; EPA: eicosapentaenoic acid; DHA: docosahexaenoic acid. The percentage (%) of fatty acids (saturated and unsaturated) was calculated according to the total FA lipid class, respectively. The comparison of means is established between groups II, III, and IV and the control group. ns, *p* > 0.05, * *p* < 0.05; ** *p* < 0.01; *** *p* < 0.001.

## Data Availability

The data presented in this study are available on request from the corresponding author.
